# Prediction and evaluation of the energy structure under the green finance development in Chongqing municipality, China^☆^

**DOI:** 10.1016/j.heliyon.2023.e22481

**Published:** 2023-11-19

**Authors:** Sheng Zeng, Yangchen Yu, Wenze Li

**Affiliations:** a Research Center for Economy of Upper Reaches of the Yangtse River, Chongqing Technology and Business University, Chongqing, China; b School of Finance, Chongqing Technology and Business University, Chongqing, China

**Keywords:** Green finance, Energy structure, Chongqing, Copula function, SSA-MFD-SVR, ARIMA model

## Abstract

Chongqing, as the last ecological barrier of the Upper Yangtze River, is constrained to achieve “dual carbon” goals due to imbalanced energy structure. Based on selecting the energy structure influencing factors through Copula function and Granger causality, a multi-dimensional dynamic support vector machine model (SSA-MFD-SVR-ARIMA) by adopting sparrow algorithm was constructed to predict the proportion of Chongqing's energy structure from 2021 to 2030 under the drive of green finance development, and an optimization path was obtained. The novel findings confirm that (1) the correlated contribution rate of Green Finance to optimizing Chongqing's Energy Structure is 10.8 %; (2) under the sustained growth rate of Green Finance at 4.5 %, the proportion of coal consumption will reach 40.03 % by 2030, and non-fossil energy consumption will account for 27 %. It confirms that Chongqing can achieve the Energy Development Plan assigned by the Central Government in 2025. The research proposes a four-dimensional optimized pathway from a financial perspective that includes green equity investments, digital finance for energy, financing environmental rights and interests, and developing an industry fund. Furthermore, our put forward the safeguard strategies for financing, innovation, linkage, and protection mechanisms of this pathway optimization.

## 1 Introduction

Global climate change is one of the most critical environmental challenges that the world is currently confronting. China, with its massive energy consumption and energy endowment characterized by abundant coal but scarce oil and natural gas, is facing increasingly severe environmental degradation and frequent extreme heatwaves. China had already become the world's largest emitter of carbon dioxide as early as 2005, accounting for one-fourth of global carbon emissions [1]. By 2020, its annual carbon dioxide emissions had reached a staggering 75 million tons [2]. As a result, the Party Central Committee of China has resolutely taken up essential responsibility in the global environmental protection framework and attached great importance to pollution reduction and carbon mitigation. At the 75th session of the United Nations General Assembly in 2020, it planned a milestone-oriented emission reduction roadmap, setting targets to strive to reach the peak of carbon dioxide emissions by 2030 and achieve carbon neutrality by 2060 [3]. The specific cases of China's energy structure adjustment to achieve these ‘dual carbon’ goals will provide powerful references for global climate governance.

**Fig. 1:**
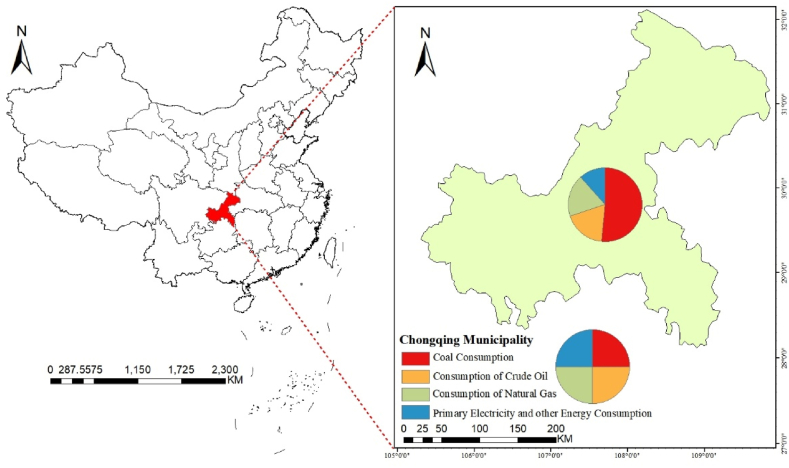
Energy consumption structure of Chongqing in 2020.

In China, Chongqing is situated in the core area of the Three Gorges Reservoir, serving as a crucial strategic hub for the development of western China, a junction point for the “Belt and Road” initiative and the Yangtze River Economic Belt. It plays a unique and crucial role in regional coordinated development of China while also focused on mitigating potentially greenhouse gas emissions. The national major regional development strategy serves the construction of beautiful cities with clear mountains and clean waters in key cities of China, including Chongqing. General Secretary Xi Jinping emphasized the need for Chongqing to play a demonstrative role in developing green initiatives of the Yangtze River Economic Belt. According to the National Bureau of Statistics of China from 2020, Chongqing had the coal consumption rate of 51.6 %, followed by oil at 18.4 %, natural gas at 18.3 %, and electricity at 11.3 % (Fig. 1).

**Fig. 2:**
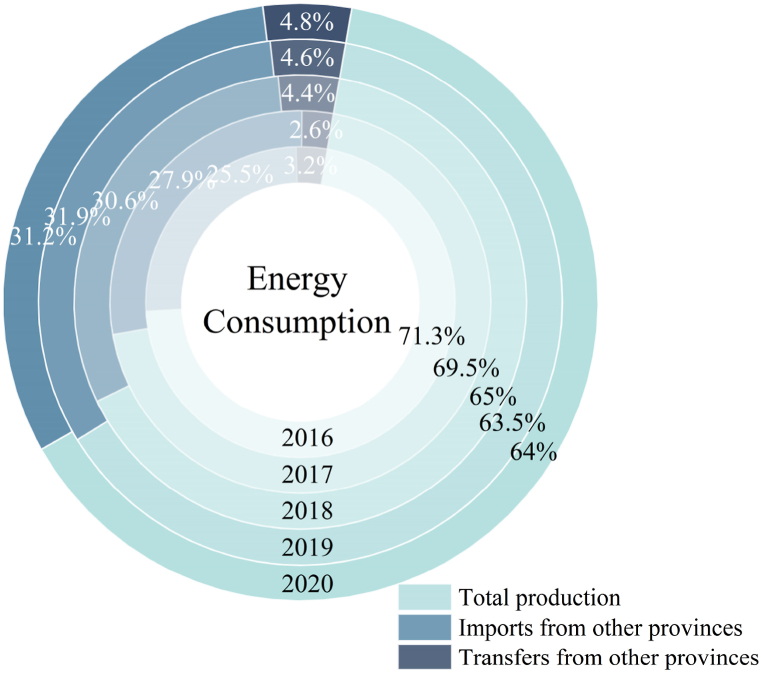
Energy consumption composition of Chongqing (2016–2020).

Chongqing is a region characterized by scarce energy resources and high energy dependence on external sources, facing critical challenges in balancing its energy supply and demand (as presented in Fig. 2).

Additionally, conventional hydropower resources have been widely exploited, renewable energy resources are limited, and the energy consumption is still predominantly reliant on coal, with less emphasis on oil, natural gas, and clean energy. As a result, this imposes substantial and formidable responsibilities on the Chongqing municipal government to support the optimization of the energy structure. In this regard, a substantial number of scholars have affirmed that this optimization should be the primary driving force that policymakers need to prioritize behind emission reduction goals as soon as possible [[4], [5], [6]]. It is crucial to enhance the development and utilization of green and low-carbon energy, as well as reduce the consumption of high-carbon energy sources like coal and oil. To achieve these objectives, the Chongqing municipal government has promulgated the “14th Five-Year” energy development plan (2021–2025), which emphasizes on non-fossil energy as the core, and aims to increase the share of non-fossil energy to 20 % by 2025 [7]. This target requires multifaceted collaboration, mutual cooperation, and joint efforts.

It is widely acknowledged in existing research that green finance plays a crucial role in ensuring energy supply and demand, transforming traditional energy production and consumption patterns, and promoting diversified development of energy consumption [[8], [9], [10]]. Moreover, green finance has been acknowledged as an innovative financial model in China's major ecological development strategy, aiming to adjust the energy structure and promote sustainable economic growth. Chongqing strives to become a demonstration city and leading pilot zone for green and low-carbon development in China. While there are numerous factors involved in improving Chongqing's energy structure, green finance will provide a unique perspective and an urgent topic for discussion in optimization of the regional energy structure in the future. In this context, previous literature has examined the causal relationship between green finance and energy structure, which confirms that green finance is a vital measure for optimizing the energy structure [8,11]. Furthermore, research on exploring the link between regional energy structure and carbon emissions has gradually attracted the attention of experts [[12], [13], [14], [15]]. Nevertheless, there is a dearth of research at the municipal level, particularly predicting energy consumption composition and exploring the optimization path of energy structure from the perspective of green finance Specifically, Chongqing holds great significance in both energy consumption and production sectors within the strategy of China's green and low-carbon development, as the pivotal city of comprehensive transportation hub in Southwest China and one of China's new first-tier cities. The most urgent issue regarding sustainability in Chongqing is how to optimize the energy structure and establish a modern and innovative energy system. This issue is unprecedented, emphasizing the urgent need for theoretical guidance and research work.

In light of this, we investigate the energy structure of Chongqing as a microcosm and a specific example to address the limitations and tackle the following issues: (1) Predicting the proportion of energy structure in Chongqing during the “14th Five-Year Plan” period (2025–2030) under the rapid development of green finance. (2) Exploring the pathway of optimizing the energy structure in Chongqing through green finance.

**Fig. 3:**
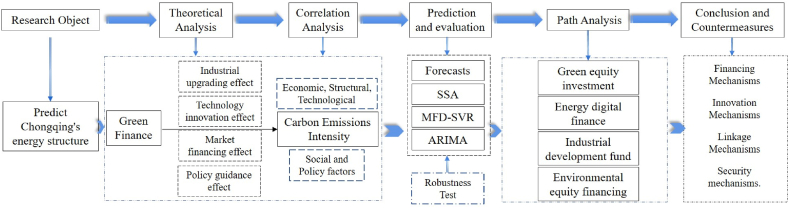
The organizational structure of research.

Therefore, this research takes the energy structure of Chongqing from 2000 to 2020 as the research object. The Copula function and Granger causality test are utilized to identify and select influential factors of energy structure, and based on the ARIMA model of time series, a multidimensional dynamic support vector machine model with the sparrow algorithm (SSA-MFD-SVR-ARIMA) is employed to predict the proportion of energy structure under the promotion of green finance from 2021 to 2030, thus obtaining the optimal path for green finance to facilitate energy structure optimization. The organizational structure of research is shown in Fig. 3. This paper makes notable contributions, specifically: (1) Enriching the research outcomes in the fields of energy economics, resource economics, and climate economics. The relevance assessment indicates that the contribution of green finance to the energy structure in Chongqing should not be overlooked. Based on relevant research [[16], [17], [18]], This research predicts the energy structure in Chongqing from the new perspective of green finance development, confirming that the development of green finance maintains a growth rate of 4.5 %. Consequently, Chongqing has achieved the target of peaking carbon emissions before 2030 and the goal of coal consumption ac-counting for 40 % of the energy development plan by 2025. (2) Constructing a novel method evaluation of integrating the dynamic characteristics of energy structure with data analysis techniques. The SSA-MFD-SVR-ARIMA model, upon the abandonment of conventional methods, remarkably enhances the prediction accuracy, accurately assessing the energy transition roadmap of mega-cities, offering novel research insights, and implementation strategies. (3) Ascertaining the mechanism of green finance in optimizing energy structure. Green finance as a key determinant of the energy structure of adjustment by promoting industrial upgrading, technological innovation, market financing, and policy regulation effects, which is consistent with the mechanism of green finance in Chongqing. (4) Obtaining the four paths for optimization in Chongqing from a green finance perspective: green equity investment, digital finance for energy, environmental equity financing and industrial development fund provide guidance towards sustainable development of regional energy. The invention of this research is to demonstrate the interdependence and correlation among energy structure and influential variables while showcasing potential opportunities for optimization and investigating the contribution of the rapid development of green finance to the optimization of energy structure. This emerging research offers empirical insights and theoretical support for the optimization of energy transition and emissions reduction in mega-cities across the global economy.

## 2 Literature review

**Fig. 4:**
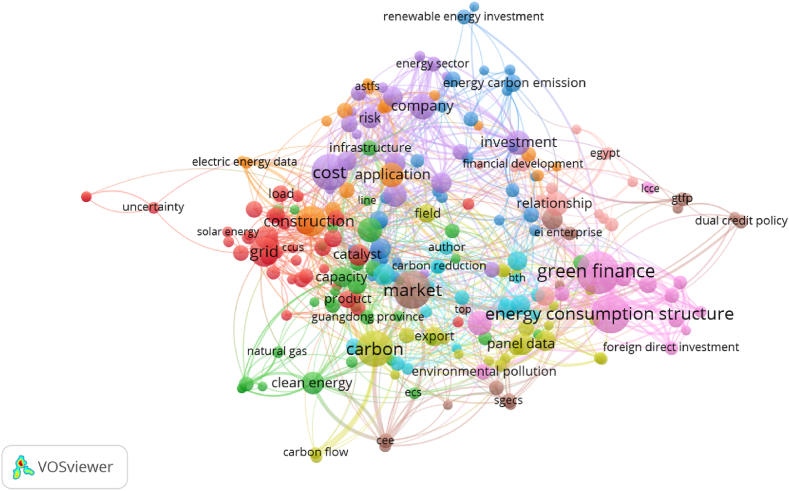
The literature visualization co-line network diagram.

Early research on energy structure primarily aimed at meeting energy consumption requirements. Both domestic and international research primarily focused on environmental pollution and fuels. Subsequently, research shifted towards the exploration of energy structure adjustments under the search for alternative energy sources. The mainstream discourse revolved around the relationship between energy structure and economic growth. Countries worldwide are pursuing the satisfaction of development requirements and stable supply-demand equilibrium. Early Chinese research proposed reforming the energy structure to eliminate atmospheric pollution. China first introduced the optimization of the energy structure and the enhancement of energy utilization efficiency in the “Tenth Five-Year Plan” in 2000. Subsequently, a substantial number of scholars concentrated on the theme of modernizing energy services provision. Using “energy structure” as a keyword, there are 20,540 papers in the Web of Science journal database. There are 1372 papers on energy structure forecasting in the field of social sciences. A total of 1613 thematic documents focus on energy structure and financial development. The literature visualization co-line network diagram is shown in Fig. 4. We have collated and summarized a large amount of relevant literature from three perspectives: energy structure optimization research, research on the “dual carbon” goals and energy structure optimization, and research on green finance and energy structure optimization, based on Fig. 4. This provides essential support for studying energy structure optimization from the standpoint of green finance.

Research on energy structure. Exogenous drivers for optimizing energy structure are factors affecting the energy structure. It is widely believed in academia that economic development, structural adjustments, technological upgrades, and policy guidance accelerate the optimization of energy structure, facilitating energy transition. The economic factor. Research indicates that there is a causal relationship between the macroeconomic total of developing countries and energy consumption in both the short and long term [19]. The variables associated with the causal relationship between energy structure and the economy do not only involve economic growth [20], but also encompass economic structure [21]. In addition, the energy prices [22], per capita GDP [23], and household income [24] constitute latent forces for optimizing energy structure. The structural element. Industrial restructuring is driving the evolution of China's energy structure [25], and as industrialization rates increase, the proportion of various energy consumption is also improving [26]. The technology factor. Technological innovation and progress contribute to the optimization of the energy structure [27]. Moreover, scholars have found that elements driven by technological changes tend to favor non-fossil fuels, thereby improving the energy structure [28]. The policy considerations. Attributable to the environmental monitoring costs, environmental regulations promote the optimization of energy consumption structure [29,30]. The social factor. It is believed that differences in energy consumption behavior among residents will affect the energy structure [31].

Energy production and consumption drive economic development. Energy supply and demand forecasting has been a focal point of academic attention since the 1980s. Empirical research on energy demand forecasting mainly utilize support vector machine models, time series forecasting models, and non-linear artificial intelligence methods. Since 2015, the majority of scholars have been constructing support vector machine models to predict energy consumption [[32], [33], [34], [35], [36]]. However, it wasn't until 2020 that experts constructed the least squares support vector machine model and particle swarm optimization algorithm to predict energy consumption structure [24]. Subsequently, multidimensional dynamic support vector machine models (MFD-SVR) were derived based on the existing foundation. By predicting China's energy consumption structure, it has been shown that the proportion of non-fossil fuels in China will reach 21 % by 2026 and peak before 2030 [16]. Furthermore, a significant amount of literature utilizes time series forecasting models to predict energy supply and demand, including ARIMA models, autoregressive distributed lag (ARDL) models, and the Long-range Energy Alternatives Planning (LEAP) model [[37], [38], [39], [40]]. China's coal-based energy consumption structure will improve by 2030, and coal will account for 55.22 % [39]. The most popular method is the use of non-linear artificial intelligence methods for energy forecasting. Scholars have used artificial neural networks and other methods to predict total energy consumption [41]. Furthermore, system dynamics models are extensively employed for forecasting and simulation of energy structures [42]. The corresponding research frequently combines models with scenario analysis methods to prognosticate the distribution of energy consumption structures induced by the substitution effects of natural gas from 2019 to 2050 and delve into its evolutionary mechanism [43]. Additionally, a new tool has been developed to predict energy consumption structure, and it has been proven that fuzzy time series models and information granularity prediction are more accurate [44].

Nations worldwide have successively proposed plans for carbon peaking and the development of carbon-neutral societies. Researchers are concentrating on forecasting energy supply and demand to attain carbon reduction objectives. Research on the “dual carbon” goals and the optimization of energy structure. Multiple objective function optimization models and scenario analysis have been combined to predict carbon emission peaks. The non-dominated sorting particle swarm optimization (NSPSO) method has been used to solve this model, and it has been shown that optimizing energy consumption structure is a necessary condition for achieving the carbon peak [45]. By utilizing Markov chain and dynamic stochastic general equilibrium (DSGE) models, it has been predicted that China can achieve its carbon intensity targets by 2030 under different economic growth scenarios of 2.2 % and 1.4 % [5]. System dynamics modeling and scenario analysis have been used to predict the energy consumption structure in Shandong Province, China. It has been shown that under the accelerated transformation scenario, the proportion of coal in the primary energy consumption in 2050 will decrease by at least 10 %. In addition, the study has further combined structural equation modeling and TOPSIS models to demonstrate that reducing economic development rates and optimizing energy consumption structures is the best scenario [46].

Research on green finance and energy structure optimization. Study on green finance and the optimization of energy structure. Since 1974, the philosophy of green development has been incorporated into the ﬁnancial ﬁeld. Germany established the world's first policy-oriented environmental bank, known as the “Ecological Bank.” Subsequently, green finance has witnessed rapid growth. The definition of green finance is the first provided. Green finance is recognized as an innovative financing mechanism for environmental protection, which is the concrete implementation of ecological finance and environmental finance [47]. It mainly supports green development through various financial methods such as green credit, green insurance, green securities, green investment, carbon emission trading markets and ESG investment funds [48], as well as helps guide optimal allocation of funds to resource protection, energy transformation, and sustainable development [[49], [50], [51]]. Regarding the intrinsic transmission mechanism of green finance in optimizing the energy structure. Scholars generally acknowledge that green finance has positive environmental effects in the context of industrial upgrading, technological advancement, market transactions, and policy guidance. In promoting industrial structure upgrading and technological progress. The role of financial development in promoting industrial structure upgrading and technological progress is discussed [52]. By improving the efficiency of capital allocation and reducing financial risks and borrowing costs, green finance provides financing for the green and environmental industries that typically have a longer investment return period [53,54], thereby affecting energy consumption and changing the energy structure. In the context of enhancing the energy market trading system. Green finance can guide the carbon market mechanism, optimize the price mechanism to allocate capital to the environmental protection industry, and internalize the negative externalities of environmental pollution [55,56]. In addition, the use of big data and cloud computing tools can help construct a smart energy system, set up an intelligent and diversified energy comprehensive management platform to allocate energy supply, and ensure energy demand [57,58]. In the support of policy guidance implementation. The role of green finance in supporting policy implementation is discussed. Government policies can guide the opening of carbon markets, supervise the capital that flows into green and environmental industries, restrict the expansion of high energy-consuming industries, and increase investment in energy conservation and environmental protection projects [55,59,60]. The information transmission function of green finance can also guide the ESG investment decisions of enterprises, enhance public understanding of energy conservation and environmental protection, and thereby change the energy demand situation [61,62].

In summary, the existing literature has explored numerous aspects of the energy structure. In the initial stages of research, qualitative analysis was predominant, primarily involving empirical judgments of the driving factors of energy structure. However, with the deepening of research and the impetus of practical issues, researchers, researchers relied on large-scale models and analytical tools to conduct more precise and reliable empirical quantitative research on energy supply and demand, development paths, optimization solutions, pollution reduction, and carbon reduction. Nevertheless, current research still exhibits certain limitations, primarily concentrated on the following areas. Firstly, although there has been considerable scholarly attention to energy consumption, production, and energy supply-demand forecasts, energy structure forecasting remains relatively scarce. Most of the research is methodological, lacking comprehensive explanations of the underlying mechanisms and specific pathways. Secondly, there is a mutual linkage between energy development and the attainment of the “dual-carbon” objectives. The consensus has been reached that optimizing energy structure is a crucial pathway to achieving these goals; however, many studies have not yet provided sufficient confirmation. Thirdly, based on current research findings, finance serves as a catalyst for energy transformation and transition. The contribution of finance to energy development cannot be overlooked. The use of green finance as a driving variable for optimizing energy structure is relatively uncommon. Moreover, literature explaining the contribution level of influencing variables is sparse, making it challenging to accurately assess the driving effect of finance on energy structure optimization. Fourth, the variables affecting energy structure possess stochastic dynamic characteristics. Dynamic variables lead to questioning the accuracy of prediction results. Additionally, linear prediction models have been extensively employed in energy consumption forecasting. Nevertheless, the energy structure is a vast and complex system, making precise predictions challenging with traditional methods. Issues like insufficient stability in prediction results and challenges in determining parameter optimization determination persist. Fifth, the search for energy optimization pathways is undertaken through energy structure predictions. Practical application still faces certain limitations. Meanwhile, research on carbon peaking in mega-cities lacks energy structure optimization pathways and plans from the financial perspective of Chongqing City.

## 3 Theoretical analysis

### 3.1 Influential mechanism of energy structure

The study examines the impact of economic, structural, technological, social, and policy factors on energy consumption structure across five dimensions.

Economic Factors. Changes in energy consumption often align with economic growth, exhibiting an upward trajectory. Key economic factors affecting energy structure include macroeconomy (G/GZ), fixed asset investment (Z), energy prices (N), per capita GDP (RG), household income (CK/NK), and green finance (GF). These factors drive energy demand, elevate energy technology standards, affect energy consumption, alter energy supply, and modify consumption habits, thus shaping energy consumption structure. Macroeconomic metrics (G/GZ) dominate energy substitution, stimulate energy demand, and propel technological advancement. Fixed asset investment (Z) effectively scales up production, yielding industrial scale effects and ultimately impacting energy consumption structure through changes in supply and demand. Energy prices (N) encompass production and final product prices, with price dynamics affecting supply and demand. Per capita GDP (RG) and household income (CK/NK) increases stimulate household energy consumption, potentially altering energy consumption habits and tendencies, thus impacting macro energy consumption structure. Green finance (GF) advances clean energy supply and demand, fostering advanced energy structure development.

Structural Factors. Industrial and consumption structure changes within the economic framework are closely tied to fossil fuel consumption. Structural factors encompass industrial structure (C), household consumption structure (CE/NX), and energy consumption structure (NX/GX/ZX). Distinct industries exhibit varying energy consumption preferences and patterns, with the tertiary industry consuming significantly less than the primary and secondary sectors. In household consumption, fossil fuel consumption is inherent. Residents' consumption inclinations and environmental awareness collectively influence the total amount and composition of energy consumption. Energy consumption structure refers to energy distribution among major sectors, and differences in production modes contribute to heterogeneous energy demand across industries.

Technological Factors. Technological innovation drives economic development and concurrently influences energy consumption and supply. Research and development capabilities in science and technology incentivize energy structure optimization. Factors encompass labor productivity (LS), energy intensity (NQ), carbon emission intensity (TQ), research and development investment (RDJ/RDG), and patent grants (K). Enhanced labor productivity can elevate industrial production efficiency, potentially increasing energy consumption. Energy and carbon intensity are intricately linked to fossil fuel consumption. Greater research and development investment supports low-carbon technology development, aiding in the reduction of fossil fuel consumption and facilitating green transformation. The number of patents granted to a company's research and development division can significantly impact its future sustainable development rate. Technology enhances efficiency, reduces energy consumption, carbon emissions, and sustains long-term business development.

Social Factors. Social development closely correlates with energy demand. Social factors include population size (R) and urbanization level (CL). Population size directly affects total energy consumption, energy resource utilization, alongside per capita consumption. Notably, energy consumption levels and usage patterns diverge substantially between urban and rural residents.

Policy Factors. These factors can be categorized into two sections: the degree of marketization (S) and the level of environmental regulation (ER). Marketization degree and government intervention have an inverse relationship. A higher degree of marketization emphasizes profit-driven motives, while lower marketization underscores administrative intervention, emphasizing energy-saving, emission reduction, and cost-conscious energy consumption.

### 3.2 Theoretical mechanism of green finance

**Fig. 5:**
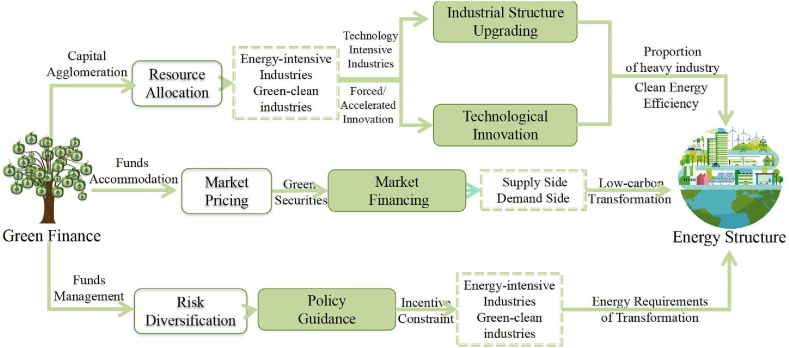
The mechanism of green finance to optimize energy structure. (For interpretation of the references to colour in this figure legend, the reader is referred to the Web version of this article.)

The mechanism by which green finance optimizes energy structure is evident through the comparison between high-energy-consuming industries, “two highs and one surplus” industries, energy-saving and environmental protection industries, and clean industries. Green finance plays a vital role in industrial upgrading, technological innovation, market financing, and policy guidance, which leads to the optimization of energy structure and promotes sustainable development. The transmission path of green finance in Chongqing is in line with the overall transmission path. To make the most of its financial resource allocation function, it is necessary to adapt to Chongqing's factor endowment, ecological characteristics, and economic scale, explore ecological compensation mechanisms across regions and river basins, promote the capitalization transformation of regional ecological resources, and establish a model for financial support for the green and low-carbon development of the Yangtze River Economic Belt and even the entire country. We also draw a mechanism diagram to illustrate the above relationships about green finance and energy structure in Fig. 5.

#### 3.2.1 Industrial upgrading effect

The green finance development policy aims to support the staged upgrading of China's industries, rapidly propel the transition of the industrial structure towards green transformation, promote the technological innovation of clean industries [63]. Green finance effectively guides financial market funds to transfer to green industries such as new energy, energy conservation, and environmental protection, while correspondingly increasing the financing costs of energy-intensive, high-pollution, and overcapacity enterprises and projects, ultimately achieving the transformation of the industrial structure towards rationalization and high-end development. With the rapid flow of capital elements in the green field, resource utilization efficiency continues to improve, and the industrial structure gradually shifts from labor-intensive to capital and technology-intensive, adjusting to a resource-saving model. Resources are shifted from highly polluting industries to service and light industries with high technological intensity and low pollution, thus reducing resource waste and driving the continuous increase of product added value and economic benefits. The optimization of the industrial structure achieves a decrease in the proportion of heavy industry and an increase in the proportion of light industry and service industry. The adjustment of the industrial structure and energy structure mutually coordinate and promote each other [64,65]. Different types of industries have differing energy supply requirements, and service industries have relatively lower demand for fossil energy than heavy industries. In addition, in the light industry, the development of industrial strategic intelligent industries, high-tech industries, and environmental protection low-carbon industries mainly relies on the mode of green and clean industries that uses new energy to promote industrial development. As the added value of the light industry and the service industry increases, the proportion of demand for fossil fuels decreases, and the proportion of demand for new energy in the energy consumption structure continues to rise. Therefore, by developing energy types such as solar energy, wind energy, and bioenergy, the proportion of coal in the energy structure can be reduced, and more industrial development can be empowered under the established coal equivalence, resulting in a more low-carbon outcome.

#### 3.2.2 Technology innovation effects

As a reservoir for green funds, finance plays a positive role in effectively guiding and marketizing green technology innovation. Its role is not limited to supporting the clean industry but rather, it is critical to open up funding channels for environmental technology upgrades in high-pollution and high-energy-consumption industries [66,67]. Green finance guides capital to flow from the polluting field to the clean field, forcing high-energy-consuming industries to innovate in low-carbon technologies and supporting the technological research and development of energy-saving industries [68]. Green finance limits financing scale for high-energy-consuming enterprises, forcing enterprises to increase their investment in production technology and emission reduction technology, while at the same time, switching to lower-carbon and more environmentally friendly production modes, thereby providing an opportunity for enterprises to achieve high-quality development. Green finance increases research and development expenditures in new energy extraction and storage, green low-carbonization, and pollution control technologies, accelerating the pace and number of green patent research, and promoting capitalization of green technologies, thereby increasing their innovation scale. The disclosure mechanism of green finance provides risk compensation and guarantee mechanisms, controlling the risk of technological innovation while saving research and development costs, facilitating risk hedging and mitigation. Technological innovation in high-energy-consuming industries improves production efficiency, reduces consumption of fossil fuels and adjusts the energy consumption structure [69]. Green-biased technological innovation plays an important role in achieving energy-saving and emission reduction in heavy industry, improving the conversion efficiency of energy structure optimization. Technological innovation in the new energy industry effectively reduces extraction costs and speeds, optimizing the energy structure predominantly composed of fossil fuels. Digital technology innovation lays the foundation for building an intelligent new energy interconnection platform, providing support for long-distance transmission and storage of electricity and promoting further development towards a lower-carbon energy future.

#### 3.2.3 Market financing effect

China's energy consumption structure, dominated by coal, determines that coal industry loans account for a relatively high proportion of all energy financing. However, as banks and other financial institutions strengthen their green loan business, green finance development has gradually matured, and market financing orientation has gradually begun to change. The expanded credit supply and the opening up of green channels provide green support for traditional enterprises that are actively transforming. Green project construction can obtain sufficient service loans; green insurance integrates the concept of green development into insurance products and services, and provides long-term stable financing support for green projects, ensuring the sustained green development of enterprises [70]. The market-oriented role also guides social capital towards the green field, with private capital participating in green development and promoting green development. The development of the green finance market has enabled funds to flow into green projects, solving the problem of difficult financing for green projects, and achieving further expansion and development, which are closely related to energy. From the supply perspective, the energy supply industry realizes the green transformation by obtaining green financing, providing clean energy supply, and reducing carbon emissions. From the consumption perspective, the energy-intensive industry also realizes the green transformation through financing, but in the process of transformation and development, it uses less energy and prioritizes the use of clean energy, thus reducing greenhouse gas emissions [71]. The green transformation of industries can further improve the energy structure. In addition, green projects absorb green funds, provide technical support for industrial transformation, and provide a supportive foundation for energy structure transformation.

#### 3.2.4 Policy guidance effect

The government plays a pivotal role as a guide and supporter in the development of green finance. On the one hand, regulations and legal provisions create a favorable external environment for the development of green finance, which further promotes the optimization of the energy structure. From the perspective of financial institutions, policies such as incentive policies have been introduced to increase the enthusiasm of financial institutions, particularly non-bank financial institutions, to participate in green projects. Examples of these policies include green loan interest subsidies, green bond interest subsidies, and risk compensation policies. At the same time, the policy of re-lending and rediscounting plays a guiding and promoting role, as seen in specialized tools such as “Green-easy Loans” and “Green Bill Discount” which support financial institutions in increasing the flow of green loans, increasing green asset allocation, and ensuring that funds flow into the green sector. This also enables energy industries facing transformation difficulties to obtain concessional loans, thereby achieving industrial upgrading. From the perspective of enterprises, the government perfects laws and policies on intellectual property protection and green innovation support, guiding enterprises to allocate more funds towards green research and development projects. Furthermore, preferential policies for new energy sources are introduced to lower the cost of using renewable energy and encourage energy consumers to shift to cleaner sources. In addition, through the government's green fiscal expenditures, a powerful booster and new engine for the green finance market is created, driving more social capital and supporting green industry development. The upgrading of industrial structure towards clean energy integration signals the shift in energy structure adjustment. Direct fiscal support of enterprises caught in the midst of green transformation, as well as tax incentives and subsidies, are offered in order to alleviate the funding difficulties involved in transformation [72]. Once enterprises make technological breakthroughs or innovative achievements in green energy, there will be significant benefits in terms of reducing traditional energy usage, increasing energy utilization efficiency, and increasing the supply of new and clean energy. This in turn promotes the optimization of the energy structure.

The government plays a supervisory and regulatory role in the development of green finance, acting as both a monitor and a source of constraints. By limiting the flow of capital from financial entities, the government effectively encourages green energy transformation. On the one hand, the government imposes pollution treatment fees and carbon taxes on traditional energy companies and high-polluting energy-consuming industries, compelling enterprises to reduce the consumption of non-renewable energy sources. Additionally, the government leverages acquired funds to support environmentally-friendly business operations, while simultaneously reducing the cost of renewable energy use. These measures can help drive the transformation of the energy structure. On the other hand, effective regulation of the green finance market prevents financial institutions from exploiting policy loopholes to direct funds towards other sectors. In terms of enterprise regulation, carbon emission costs are raised for high-energy-consuming and high-polluting enterprises, while strict punishment is imposed on enterprises that engage in illegal pollution discharge. Restrictive policies and effective regulation promote the development of the green industry, encourage energy-consuming enterprises to adopt renewable energy sources, and accelerate the transformation and upgrading of the energy structure.

## 4 Influencing factors of energy structure and correlation analysis

This investigation measures the energy consumption structure through the index of energy structure sophistication. The index of energy structure sophistication can be expressed as the ratio of total electricity consumption and natural gas consumption to total coal consumption [73], as in Equation (1):

(1) $$NY=\frac{D+T}{M}$$

Here, D represents total electricity consumption, T represents total natural gas consumption, and M represents total coal consumption. If the NY value is in an upward trend, it indicates that the energy structure is in an optimized phase; otherwise, it suggests that the energy structure is deteriorating and irrational.

### 4.1 Selection and measurement of variables

Drawing upon the unique geographical location and economic development model of Chongqing, researchers refer to studies on energy supply, demand, and structural forecasts to select influential factors from five dimensions: economic, structural, technological, social, and policy factors. The energy structure evaluation system consists of five primary indicators [74], 18 secondary indicators, and 26 characterization indicators, as illustrated in Table 1. The significant innovation of this paper lies in considering the contributions of green finance and environmental regulations as factors influencing the energy structure.

**Table 1:** Indicators of factors influencing energy structure.

Level 1 Indicator	Level 2 Indicator	Representing Indicator	Indicator Representation
Economic Factors	Macroeconomics	Gross Domestic Product	G
		Economic Growth Rate	GZ
	Level of Fixed Asset Investment	Overall Level of National Fixed Asset Investment	Z
	Energy Prices Price	Energy Prices Index for Raw Materials, Fuel, and Power	N
	Per Capita GDP	Per Capita GDP	RG
	Green Finance	Green Finance Comprehensive Index	GF
	Level of Household Income	Per Capita Disposable Income of Urban Residents	CK
		Per Capita Disposable Income of Rural Residents	NK
Structure Factors	Industrial Structure	Ratio of Value Added of Tertiary Industry to Secondary Industry	C
	Residents Consumer Structure	Engel's Coefficient of Urban Residents	CE
		Engel's Coefficient of Rural Residents	NE
	Energy Consumption Structure	Proportion of Energy Consumption in Agriculture, Forestry, Animal Husbandry, and Fishery	NX
		Proportion of Energy Consumption in Industry	GX
		Proportion of Energy Consumption in Manufacturing	ZX
		Proportion of Energy Consumption in Daily Life	SX
Technological Factors	Labor Productivity	Overall Labor Productivity	LS
		Energy Consumption per Unit of GDP	NQ
	Energy Intensity Carbon Intensity	Carbon Emissions per Unit of GDP	TQ
	R&D Investment	R&D Expenditure	RDJ
		Proportion of R&D in GDP	RDG
		R&D Expenditure on Scientific	K
	Patent Grants	Number of Granted Patents	ZS
Social Factors	Population Scale	Total Population	R
	Urbanization Level	Urbanization Rate	CL
Policy Factors	Level of Marketization	Marketization Rate	S
	Environmental Regulation	Comprehensive Index of Environmental Regulation	ER

Green finance will be established from five dimensions: green credit, green insurance, green investment, green securities, and carbon finance, creating a comprehensive evaluation system with nine indicator levels (as presented in Table 2.). The comprehensive index of green finance will then be measured using the entropy method [75]. Relevant indicators are explained as follows: Green credit indicators. The interest expense ratio of high energy-consuming industries is selected as the green credit indicator, which reflects the support of financial institutions in green industries and the restraint in high pollution and high energy-consuming industries. The indicator of the proportion of green credit scale directly manifests the regional green credit scale structure by the ratio of loan balance of energy-saving and environmental protection enterprises to the loan balance of financial institutions [76]. Green securities indicators. The market value ratio of environmental protection enterprises and high energy-consuming industries are employed to indicate the financing of various types of enterprises in the securities market. Green insurance indicators. The proportion of agricultural insurance scale and agricultural insurance payout are applied to estimate the level of regional green insurance development. Agricultural insurance was selected as a proxy variable for environmental pollution liability insurance, because there is no authoritative inter-provincial data on environmental pollution liability insurance in 2013 and the strong dependence of agriculture on the natural environment. Green investment indicators. The percentage of environmental protection investment and the percentage of green expenditure are applied to gauge green investment reflecting the capacity of green investment in sustainable development. Carbon finance indicators. The carbon emission depth indicator is chosen to quantify the loan intensity of regions carbon emission because of the serious lack of data of CDM projects [77].

**Table 2:** Green finance evaluation index system.

Target layer	Indicator layer	Criteria layer	Indicator meaning	Attributes
Green financial development index	Green credit	Ratio of interest expenditure of high energy-consuming industries	Interest expenditure of six high energy-consuming industrial industries/total interest expenditure of industrial industries	–
		Ratio of green credit scale	Green credit balance/financial institution loan balance	+
	Green securities	Ratio of market value of environmental protection enterprises	Market value of A-share of environmental protection enterprises/total market value of A-share of listed enterprises	+
		Ratio of market value of high energy-consuming industries	Market value of A -share of high energy-consuming enterprises/total market value of A-share of listed enterprises	–
	Green Insurance	Ratio of agricultural insurance scale (Environmental liability insurance)	Agricultural insurance volume/total insurance	+
		Agricultural insurance payout ratio (Environmental liability insurance)	Agricultural insurance expenditure/Agricultural insurance income	+
	Green investment	Ratio of environmental protection investment	Investment in environmental pollution control/GDP	+
		Ratio of green expenditure	Fiscal expenditure on green industry/total fiscal expenditure	+
	Carbon finance	Carbon emission depth	Local and foreign currency loan balance/CO2 emissions	+

The original data is obtained from the “China Statistical Yearbook” for the years 2000–2020, the National Bureau of Statistics website [78], the “China Energy Statistical Yearbook” (original data for total energy consumption and consumption of various types of energy) [79], the Chongqing Municipal Bureau of Statistics website [80], and the WIND database. Furthermore, the regional gross domestic product (GDP) indicator underwent comparability adjustments using the GDP chain index with the base year of 2000, and subsequent processing was performed after obtaining comparable value indicators. Some missing data were imputed using interpolation methods. To ensure data stability and overcome heteroscedasticity, all data is transformed into logarithmic yield sequences.

### 4.2 Analysis of variable dependence

Selecting Copula functions as the method for measuring variable dependence [81]. Initially, based on data that have passed tests for stationarity, cointegration, and Granger causality, five function models including normal Copula, t-Copula, Gumbel Copula, Clayton Copula, and Frank Copula are constructed and solved. Subsequently, the Kendall rank correlation coefficient, Spearman rank correlation coefficient, and tail correlation coefficient are computed. Finally, the Euclidean squared distance test is employed to determine the most suitable Copula function. The tail correlation value of the most appropriate Copula function is used as a measure of variable interdependence, aiding in the selection of variables with high contributions to the energy structure.

#### 4.2.1 Brief description of Copula Function models

Definition of Copula Functions and Sklar's Theorem. Copula functions, denoted as C, typically adhere to several conditions: the domain of function is $I^{n}={\left[0,1\right]}^{n}$ ; C is grounded and n-dimensional increasing; the marginal distributions of C is $C_{k}$, satisfying where $C_{k}\left(u\right)=\left(1,\ldots ,1,u,1,\ldots 1\right)=u$, k=1,…n*,* u∈I.

(1) Gaussian Copula Function, as in Equation (2)

(2) $$C\left(u,v\right)=\int_{-\infty }^{\Phi^{-1}\left(u\right)}\int_{-\infty }^{\Phi^{-1}\left(v\right)}\frac{1}{2\pi \sqrt{1-r^{2}}}\exp\left\{-\frac{x^{2}-2rxy+y^{2}}{2\left(1-r^{2}\right)}\right\}dxdy$$

where u, v are related variables to be compared, r is the linear correlation coefffcient r∈[−1,1], and $\Phi^{-1}\left(\cdot \right)$ is the inverse function of the standard normal distribution function.

(2) T-Copula Function, as in Equation (3)

(3) $$C^{Ga}\left(u,v:\rho ,k\right)=\int_{-\infty }^{k^{-1}\left(u\right)}\int_{-\infty }^{k^{-1}\left(v\right)}\frac{1}{2\pi \sqrt{1-\rho^{2}}}\exp\;{\left(-\frac{s^{2}-2\rho st+t^{2}}{k\left(k\left(1-\rho^{2}\right)\right)}\right)}^{-\left(k+2\right)/2}dsdt$$

where the linear correlation coefffcient ρ is subjected to −1≤ρ≤1. $k^{-1}\left(\cdot \right)$ is the inverse function of the one-variable t distribution function $k^{-1}\left(\cdot \right)$, and both functions have the degrees of freedom of k.

(3) Gumbel Copula Function, as in Equation (4)

(4) $$C\left(u,v\right)=\exp\left\{-{\left[{\left(-\ln\;u\right)}^{\delta }+{\left(-\ln\;v\right)}^{\delta }\right]}^{1/\delta }\right\}$$

where δ≥1.

(4) Clayton Copula Function, as in Equation (5)

(5) $$C\left(u,v\right)={\left(u^{-\delta }+v^{-\delta }-1\right)}^{-1/\delta }$$

where δϵ[0,∞].

(5) Frank Copula Function, as in Equation (6)

(6) $$C\left(u,v\right)=-\frac{1}{\delta }\ln\left(\left[\eta -\left(1-e^{-\delta \nu }\right)\left(1-e^{-\delta \nu }\right)\right]/\eta \right)$$

where δϵ[0,∞], $\eta =1-e^{-\delta }$.

Correlation metrics. We apply the Kendall rank correlation coefffcient, Spearman rank correlation coefffcient and tail correlation coefffcient in extreme cases to choose the correlation between variables and form correlation metrics.

Kendall rank correlation coefffcient, as in Equation (7)

(7) $$\tau =P\left[\left(u_{1}-u_{2}\right)\left(v_{1}-v_{2}\right)>0\right]-P\left[\left(u_{1}-u_{2}\right)\left(v_{1}-v_{2}\right)<0\right]$$

where $u_{i}$ and $v_{i}$ are two sets of variable data.

Spearman rank correlation coefffcient, as in Equation (8)

(8) $$\rho =3\left\{P\left[\left(u_{1}-u_{2}\right)\left(v_{1}-v_{3}\right)>0\right]-P\left[\left(u_{1}-u_{2}\right)\left(v_{1}-v_{3}\right)<0\right]\right\}$$

Upper and Lower Tail correlation coefffcient, as in Equations (9), (10)

(9) $$\lambda^{U}=\lim_{\alpha \to 1^{-}}P\left[V>F_{2}^{-1}\left(a\right)|U>F_{1}^{-1}\left(a\right)\right]$$

(10) $$\lambda^{L}=\lim_{\alpha \to 0^{+}}P\left[V<F_{2}^{-1}\left(a\right)|U<F_{1}^{-1}\left(a\right)\right]$$

Assuming that there are random variables U and V with corresponding marginal distribution functions as F1(U), F2(V).

Where. $F_{1}^{-1}\left(a\right)=\inf\left(u|U\geq a\right)$, $F_{2}^{-1}\left(a\right)=\inf\left(v|V\geq a\right)$.

Model evaluation. We utilize the square Euclidean distance method to assess the quality of the goodness of fitting applied to the constructed Copula function model. The empirical Copula function can be expressed as in Equation (11):

(11) $$\overset{\wedge }{C_{n}}\left(u,v\right)=\frac{1}{n}\sum_{i=1}^{n}I_{\left[F_{n}\left(x_{i}\right)\leq u\right]}I_{\left[G_{n}\left(y_{i}\right)\leq v\right]},u,v\in \left[0,1\right]$$

where the set (xi,yi) (i = 1,2, …,n) represents a sample drawn from a two-dimensional population (X, Y). $F_{n}\left(x\right)$ and $G_{n}\left(y\right)$ denote the empirical distribution functions of X and Y, respectively. I(⋅) represents the indicator function. The squared Euclidean distance between the target Copula function and the empirical Copula can be expressed as in Equation (12):

(12) $$d^{2}=\sum_{i=1}^{n}\left|{\hat{C}}_{n}\left(u_{i},v_{i}\right)-\hat{C}\left(u_{i},v_{i}\right)\right|$$

Small d denotes a good fit. The corresponding Copula function is appropriate for calculating the tail correlation coefficient to assess the degree of correlation. The shorter the squared Euclidean distance obtained, the better the effect expressed by the Copula function, indicating a superior fit. The Kendall rank correlation coefficient and Spearman rank correlation coefficient of Copula are closest to the original sample data, and they also have the smallest squared Euclidean distance in the test with the squared Euclidean distance of the empirical Copula. This indicates that the selected Copula is better. If the rank correlation coefficient obtained is closest to the original data, but the squared Euclidean distance is not the smallest, then when selecting the optimal Copula model, it should be chosen based on the smallest squared Euclidean distance.

#### 4.2.2 Stationarity, cointegration, and granger causality test

The descriptive statistical characteristics of the sample data for each variable are depicted in Appendix A. Table A1.

The variable data is subjected to an ADF unit root test for stationarity, and the test results are presented in Appendix A. Table A2.

Based on n Table A2, the test results confirm that all data is stationary after the unit root test. Each-stage data is a stationary time series, forming the conditions for cointegration tests. The Granger causality test is the most commonly used econometric method for investigating causal relationships between two stationary sequence variables.

In addition, cointegration tests need to be conducted between the energy structure and each variable separately, and the results are shown in Table A3. Assuming two variable sequences {X} and {Y}, if adding variable X improves the prediction of the {Y} variable sequence more than using variable Y alone, it is considered that X has a Granger causal relationship with Y; otherwise, there is no Granger causal relationship between X and Y. In this context, we perform Granger causality tests on the energy consumption structure (NY) and various influencing factors. Traditional econometric methods merely qualitatively analyze the mutual relationship between the two, without accurately assessing the extent of their interdependence. Copula function measurements yield more accurate results than traditional coefficients. Therefore, this paper utilizes Copula function measurements to assess interdependence between variables, offering a fresh perspective for scholars interested in interdependence research.

**Table 3:** Tail correlation coefficients between energy structure and the influencing factors.

Variables	The optimal function	The tail correlation	The tail correlation coefficient of the optimal Copula function
NY&G	Gaussian Copula	Symmetry of tail	1.83 %
NY&GZ	Clatyon Copula	Lower tail correlation	0.01 %
NY&Z	Clatyon Copula	Lower tail correlation	1.83 %
NY&N	T Copula	Symmetry of tail	22.87 %
NY&RG	Gaussian Copula	Symmetry of tail	0
NY&GF	T Copula	Symmetry of tail	10.80 %
NY&CK	Gaussian Copula	Symmetry of tail	19.70 %
NY&NK	Clatyon Copula	Lower tail correlation	14.09 %
NY&C	Gumbel Copula	Upper tail correlation	23.60 %
NY&CE	Gumbel Copula	Upper tail correlation	8.88 %
NY&NE	Gumbel Copula	Upper tail correlation	2.88 %
NY&NX	Gumbel Copula	Upper tail correlation	15.38 %
NY&GX	Gaussian Copula	Symmetry of tail	0
NY&ZX	Frank Copula	Tail independent	0
NY&SX	Gumbel Copula	Upper tail correlation	8.41 %
NY&LS	Frank Copula	Tail independent	0
NY&NQ	Gaussian Copula	Symmetry of tail	15.71 %
NY&TQ	Clatyon Copula	Upper tail correlation	12.48 %
NY&RDJ	T Copula	Symmetry of tail	11.12 %
NY&RDG	Gaussian Copula	Symmetry of tail	0.04 %
NY&K	Gaussian Copula	Symmetry of tail	32.91 %
NY&ZS	T-Copula	Symmetry of tail	11.42 %
NY&R	T-Copula	Symmetry of tail	8.07 %
NY&CL	Frank Copula	Tail independent	0
NY&S	Gaussian Copula	Symmetry of tail	9.41 %
NY&ER	Clatyon Copula	Lower tail correlation	23.96 %

The results in Table A3 indicate the presence of long-term equilibrium relationships between variables and satisfying the conditions of the Granger causality test. The results of the Granger causality test are illustrated in Table A4.

**Table 4:** Correlation between energy structure and influencing factors.

Primary Indicator	Secondary Indicator	Representation	Correlation	Magnitude of Correlation
Economic Factors	Macroeconomics	G	–	0 or negligible
		GZ	–	0 or negligible
	Level of Fixed Asset Investment	Z	–	0 or negligible
	Energy Prices Price	N	+	22.87 %
	Per Capita GDP	RG	–	0 or negligible
	Green Finance	GF	+	10.80 %
	Level of Household Income	CK	–	19.70 %
		NK	–	14.09 %
Structure Factors	Industrial Structure	C	+	23.60 %
	Residents Consumer Structure	CE	+	8.88 %
		NE	+	2.88 %
	Energy Consumption Structure	NX	+	15.38 %
		GX	–	0 or negligible
		ZX	–	0 or negligible
		SX	+	8.41 %
Technological Factors	Labor Productivity	LS	–	0 or negligible
	Energy Intensity	NQ	+	15.71 %
	Carbon Intensity	TQ	–	12.48 %
	R&D Investment	RDJ	+	11.12 %
		RDG	+	0 or negligible
		K	–	32.91 %
	Patent Grants	ZS	+	11.42 %
Social Factors	Population Scale	R	–	8.07 %
	Urbanization Level	CL	–	0 or negligible
	Level of Marketization	S	–	9.41 %
Policy Factors	Environmental Regulation	ER	+	23.96 %

The research reveals that the fluctuations in the energy consumption structure are influenced by factors such as raw materials, fuel, power purchase price index, urban residents' Engel coefficient, and rural residents' Engel coefficient, while factors such as green finance, the ratio of the tertiary industry value to the secondary industry value, and energy consumption per unit of GDP contribute to the fluctuations in the energy consumption structure. Consequently, it is found that introducing green finance provides a higher degree of fit than using the autoregressive sequence of the energy structure alone, and further investigation is needed to understand the contribution of green finance to optimizing the energy structure. Although all other factors have long-term equilibrium relationships with the proportion of energy structure, there is no mutual causal relationship among them. The measurement of Copula functions yields more accurate results compared to traditional coefficients. Therefore, this paper adopts Copula functions to measure the interdependence between variables.

Conventional econometric methods merely qualitatively analyze the mutual relationship between the two and cannot accurately assess the magnitude of their interdependence. Measurements using the Copula function provide results that are more precise than those derived from traditional coefficients. Therefore, this paper utilizes measurements with the Copula function to assess interdependence among variables, offering fresh insights for scholars interested in interdependence research.

Basic statistical analysis is conducted on the logarithmic return series of each indicator. The practical significance of statistical analysis is to provide preliminary consideration for the subsequent selection of a more suitable Copula function. The results are obtained in Table A5.

**Table 5:** Evaluation of predictive performance on the testing set.

	RMSE	R^2^	MAE	MBE	MAPE
Gaussian Kernel SVR	0.0300	0.8345	0.0254	0.0210	5.6151
Polynomial Kernel SVR	0.0979	0.7719	0.0824	0.0343	17.4413
Linear Kernel SVR	0.0416	0.6803	0.0351	0.0344	8.5323
Sigmoid Kernel Function	0.1558	0.3490	0.1223	0.0413	45.1391
ARIMA Model	0.0572	0.5386	0.0446	0.0383	10.6105

The table shows that the kurtosis of the logarithmic returns for 12 indicators is greater than three, indicating a significant presence of leptokurtic heavy-tailed effects in the return series, including Energy Structure (NY), Green Finance Comprehensive Index (GF), Economic Growth Rate (GZ), Energy Prices Index for Raw Materials, Fuel, and Power(N), Engel's Coefficient of Urban Residents (CE), Engel's Coefficient of Rural Residents (NE), Proportion of Energy Consumption in Agriculture, Forestry, Animal Husbandry, and Fishery (NX), Overall Labor Productivity (LS), R&D Expenditure (RDJ), Proportion of R&D in GDP (RDG), Urbanization Rate (CL), Comprehensive Index of Environmental Regulation (ER).

In terms of skewness, 15 indicators have skewness values less than 0, indicating a left-skewed distribution, including Gross Domestic Product (G), Green Finance Comprehensive Index (GF), Per Capita GDP (RG), Per Capita Disposable Income of Urban Residents (CK), Per Capita Disposable Income of Rural Residents (NK), Ratio of Value Added of Tertiary Industry to Secondary Industry (C), Engel's Coefficient of Rural Residents (NE), Proportion of Energy Consumption in Daily Life (SX), Energy Consumption per Unit of GDP (NQ), Carbon Emissions per Unit of GDP (TQ), R&D Expenditure (RDJ), Proportion of R&D in GDP (RDG), R&D Expenditure on Scientific (K), Urbanization Rate (CL), Marketization Rate (S) have skewness values greater than 0, indicating a right-skewed distribution. Indicators Energy Structure (NY), Gross Domestic Product (G), Overall Level of National Fixed Asset Investment (Z), Per Capita Disposable Income of Urban Residents (CK), Per Capita Disposable Income of Rural Residents (NK), Ratio of Value Added of Tertiary Industry to Secondary Industry (C), Engel's Coefficient of Urban Residents (CE), Proportion of Energy Consumption in Agriculture, Forestry, Animal Husbandry, and Fishery (NX), Proportion of Energy Consumption in Industry (GX), Proportion of Energy Consumption in Manufacturing (ZX), Number of Granted Patents (ZS), Overall Labor Productivity (LS), Total Population (R), Comprehensive Index of Environmental Regulation (ER).

Normality tests are conducted for each indicator. For Proportion of R&D in GDP (RDG) and Proportion of Energy Consumption in Agriculture, Forestry, Animal Husbandry, and Fishery (NX), the Jarque-Bera test, Kolmogorov-Smirnov test, and Lilliefors test all have h values of 1 and p values close to 0, indicating that these indicators do not follow a normal distribution. For Gross Domestic Product (G), Green Finance Comprehensive Index (GF), Economic Growth Rate (GZ), R&D Expenditure (RDJ), Urbanization Rate (CL), and Comprehensive Index of Environmental Regulation (ER), only one of the tests in the Jarque-Bera test, Kolmogorov-Smirnov test, and Lilliefors test rejects the null hypothesis, suggesting non-normality. The remaining indicators pass the Jarque-Bera test, Kolmogorov-Smirnov test, and Lilliefors test, indicating adherence to the normal distribution.

Five Copula models are used to measure the correlation between the energy structure sophistication index and 26 influencing factor indicators, as well as the calculation of Euclidean distance, as depicted in Appendix A. Table A6.

**Table 6:** The predicted values of various influencing variables on energy structure.

Year	GF	NQ	TQ	RDJ	R	CK	NK
2021	0.164964	0.459614	0.762075	5272114	3414.66	42606.39	17424.47
2022	0.172387	0.447204	0.73464	5276332	3435.15	45375.81	18557.06
2023	0.180144	0.43513	0.708193	5280553	3455.76	48325.23	19763.26
2024	0.188251	0.423381	0.682698	5284778	3476.49	51466.37	21047.88
2025	0.196722	0.41195	0.658121	5289005	3497.35	54811.69	22415.99
2026	0.205575	0.401239	0.634429	5293237	3518.34	58374.45	23873.03
2027	0.214826	0.390807	0.611589	5297471	3539.45	62168.79	25424.77
2028	0.224493	0.380646	0.589572	5301709	3560.68	66209.76	27077.38
2029	0.234595	0.370749	0.568348	5305950	3582.05	70513.39	28837.41
2030	0.245152	0.36111	0.547887	5310195	3603.54	75096.76	30711.85

The goodness of fit of the model is examined using the squared Euclidean distance algorithm, and the Copula function with the minimum distance of the empirical distribution function is regarded as the optimal Copula function.

The tail correlation values of each variable are calculated to quantify the dependence between the energy structure and the 26 influencing factors, as detailed in Table 3.

Based on the characteristics of the elliptical Copula function and Archimedean Copula function, the tail correlation of each function is measured to explain the probability of one variable changing due to the changes in another variable. Tail correlation quantifies the likelihood of one economic variable increasing or decreasing in tandem with another economic variable. Gaussian-Copula is less responsive to alterations in tail correlation between variables, whereas t-Copula's tail correlation coefficients effectively capture the interplay between tails. The Frank Copula assumes tail independence, with a tail correlation of zero. Archimedean Copula functions exhibit asymmetric tail dependence, with the most common being the Clayton Copula function and the Gumbel Copula function. The Gumbel Copula function is highly responsive to alterations in upper tail dependence among assets, whereas the Clayton Copula function is particularly sensitive to variations in lower tail dependence among assets. Consequently, we calculate the tail correlation using the optimal Copula function between variables, with the correlation reflecting the relationship between variables and the energy structure.

As shown in the table, firstly, the correlation between energy structure and GDP (G), GDP growth rate (GZ), total social fixed asset investment (Z), per capita GDP (RG), rural Engel coefficient (NE), proportion of manufacturing industry energy consumption (ZX), R&D as a proportion of GDP (RDG), proportion of industrial energy consumption (GX), labor productivity (LS), and urbanization rate (CL) is either zero or negligible. Secondly, in terms of the ranking of correlation strength, the top 5 factors associated with energy structure are research and development (R&D) financial expenditure (K), environmental regulations (ER), industrial structure (C), and energy prices (N), with tail correlation coefficients of 32.91 %, 23.96 %, 23.60 %, 22.87 %, and 19.70 % respectively. Thirdly, the correlation between green finance (GF) and energy structure exceeds 14 variables. Moreover, as mentioned earlier, it has been demonstrated that green finance has a Granger causality relationship with energy structure in Chongqing. This paper believes that the contribution of green finance to energy structure should not be ignored, and studying the optimization path of green finance for energy structure is essential. The dependence between the advanced energy structure index and the related variables is presented in Table 4.

Based on Table 4, 16 out of 26 influencing factors exhibit a certain degree of correlation with energy structure. The selected 16 indicators are used as input variables for the multi-input single-output support vector machine model, laying the foundation for subsequent predictions.

## 5 Prediction and evaluation

Energy structure indicators primarily consist of aggregate data and are influenced by multiple factors, characterized by intricate interrelationships among these factors. For instance, time series models used for prediction often overlook the interdependencies and equilibrium between energy supply and demand. Causal models and proportional relationships have been employed to evaluate the inherent correlations within energy structure. Prediction results often suffer from overfitting due to the fluctuating characteristics of future values of correlated variables. Additionally, while neural networks in artificial intelligence models excel at addressing volatility prediction, they are most effective when working with large sample sizes. Given the limited availability of historical data for predicting Chongqing's energy structure, achieving sufficient learning and demonstrating the models' effectiveness pose significant challenges. Therefore, considering the complexity of energy structure prediction and conducting a comprehensive model evaluation, this study opts for the SSA-MFD-SVR-ARIMA model to predict nonlinear relationships and complement existing research methods.

Utilizing historical data from 2000 to 2020 in Chongqing municipality, this research focuses on the targeted prediction of the advanced index of the energy consumption structure. The prediction is carried out under the constraints of five dimensions: economy, structure, technology, society, and policy, encompassing 16 variables. The steps of training and predicting the energy structure of Chongqing Municipality based on multi-dimensional dynamic support vector machine model of Sparrow algorithm (SSA-SVR) are as follows: (1) Based on the previous screening of 16 highly correlated factors influencing Chongqing's energy structure, this chapter constructs a multidimensional dynamic support vector machine model using the sparrow algorithm. The penalty parameter is optimized through grid search and the sparrow algorithm to achieve the best predictive performance for the support vector machine regression model through training and testing. (2) Based on the “14th Five-Year Plan” outline of Chongqing municipality, this study categorizes the 17 variables into controlled and non-controlled variables. While ensuring sustainable economic development, the growth rates of the controlled variables are determined. The ARIMA model is employed to predict the values of the non-controlled variables, resulting in the final predictions of the 16 variables for the period from 2021 to 2030. (3) Utilizing the well-trained prediction model, this research predicts the numerical values of Chongqing's energy consumption structure's advanced index from 2021 to 2030 based on the 16 influencing factors. Moreover, it forecasts the proportion of coal consumption that complies with the constraints of natural gas and electricity consumption, analyzing the peak situation in Chongqing in 2030.

### 5.1 Overview of support vector machine models

Support Vector Regression (SVR), proposed in 1995, seeks the optimal trade-off between model complexity and learning ability based on limited sample information [81]. It has unique advantages in addressing challenges such as small sample sizes, nonlinearity, and high dimensionality, exhibiting accurate learning and generalization capabilities. It is commonly utilized for handling regression prediction and similar problems [82].

In SVR, the training sample data x is mapped to a high-dimensional feature space through a nonlinear mapping Ф, and then a linear model is established in this feature space to estimate the regression function. The formula is as follows:

(13) f(x)=w∙φ(x)+b

in equation (13), w represents the weight vector, and b represents the threshold. The ε-insensitive loss function is introduced and defined as follows:

(14) $${\left|y-f\left(x\right)\right|}_{\varepsilon }=\left\{ \begin{array}{c} 0,|y-f\left(x\right)|\leq \varepsilon \\ \left|y-f\left(x\right)\right|-\varepsilon ,\left|y-f\left(x\right)\right|>\varepsilon \end{array}\right.$$

in equation (14), f represents the decision function, and f(x) represents the training sample. The insensitive loss function, also known as the ε-insensitive loss function, considers the predicted value's loss as zero if the difference between the target value y and the predicted value f(x) of the regression estimation function, obtained through learning, is smaller than ε, even if they are not equal. The primary characteristic of this loss function is to provide a region without any loss for the decision function, known as the ε-tube. As the main losses come from sample points outside the ε-tube, the SVR algorithm treats the sample points within and outside the ε-tube as support vectors (SV), assisting the decision function in making decisions. The adopted support vector regression method is known as ε-SVR, and its constrained optimization problem can be expressed as:

(15) $$\min\left[\frac{1}{2}{\left\|w\right\|}^{2}+C\sum_{i=1}^{t}\left(\xi_{i}+\xi_{i}^{*}\right)\right]$$

$$s.t.y_{i}-w∙\varphi \left(x_{i}\right)-b_{i}-\varepsilon \leq \xi_{i}$$

$$w∙\varphi \left(x_{i}\right)+b_{i}-y_{i}-\varepsilon \leq \xi_{i}^{*}$$

$$\xi_{i},\xi_{i}^{*}\geq 0\text{,}i=1,2\text{,}\ldots ,l$$

In equation (15), ${\left\|w\right\|}^{2}$ controls the model complexity, $\xi =\left(\xi_{1},\xi_{1}^{*},\ldots \xi_{i},\xi_{i}^{*}\right)$ is the slack variable, and C > 0 is the penalty parameter or regularization parameter. A larger C value indicates a greater penalty for data points that fall outside the ε-tube, and it determines the balance between the smoothness of the function and the number of sample points with function value errors exceeding the ε-tube. Minimizing $\min\frac{1}{2}{\left\|w\right\|}^{2}$ means minimizing the VC dimension and using the training error as a constraint in the optimization problem, resulting in a regression function with good generalization capability.

The above optimization problem can be transformed into a dual problem by introducing a Lagrange function and solved as follows.

(16) $$f\left(x\right)=\sum_{i=1}^{N}\left(\alpha_{i}-\alpha_{i}^{*}\right)K\left(x_{i},x_{j}\right)+b$$

In equation (16), $\alpha_{i}$, $\alpha_{i}^{*}$ (i = 1,2, …,l) are Lagrange multipliers, N is the number of support vectors, is the kernel function. Based on the characteristics of the data, support vector machines can be classified into three types: linearly separable, linearly inseparable, and non-linear classification support vector machines. Different types of support vector machines have different optimization methods. Energy structure prediction in Chongqing is a non-linear problem, hence the need for a kernel function. There are various types of kernel functions, ranging from simple linear kernel functions to complex polynomial kernel functions. Choosing an appropriate kernel function for model construction can effectively address practical problems. The selection of the kernel function involves empirical judgment or multiple comparisons. For the energy structure prediction in Chongqing, we plan to use the Gaussian kernel function, which belongs to the three common kernel functions (linear kernel function, polynomial kernel function, Gaussian kernel function).

(17) $$K\left(x_{i},x_{j}\right)=\exp\left\{-{\left|x_{i}-x_{j}\right|}^{2}/\left(2\sigma^{2}\right)\right\}=\exp\left(-g{\left\|x_{i}-x_{j}\right\|}^{2}\right)$$

In equation (17), g > 0 is the kernel parameter. By controlling the hyperparameters regularization parameter C, insensitive parameter ε, and kernel parameter g, the generalization ability of support vector machines can be controlled. C is the parameter for adjusting model complexity and empirical risk, the choice of ε affects the accuracy of the regression estimation model, and g reflects the distribution characteristics of the training data samples. In order to achieve better regression performance of the SVM model, it is necessary to set relevant tuning parameters, among which the most important are the penalty parameter C and the kernel function parameter g. Currently, in practical applications, the determination methods for hyperparameters mainly include empirical determination and grid search. Grid search means first limiting the range of the penalty function C to $2^{8}$ to $2^{-8}$, and the parameter in the Gaussian kernel function is also limited to $2^{8}$ to $2^{-8}$. C and δ form the horizontal and vertical axes, and an appropriate step size is set to ultimately find the optimal parameter combination. This study will adopt the 5-step search algorithm and the sparrow algorithm (SSA) to optimize C and g, in order to obtain the optimal C and g for the regression model in practical situations.

### 5.2 Data training and model prediction accuracy analysis

Following the conventional division method of training and testing sets, this study randomly selected 15 years from the sample data of 21 years from 2000 to 2020 as the training set to train the model, and used the remaining 6 years of data as the testing set to test the predictive performance of the trained model. (1) Utilizing the training set, a Gaussian kernel SVR model is constructed for training. (2) Considering the impact of different parameter settings in SVR on the model prediction results, this study uses grid search method and sparrow algorithm (SSA) to fine-tune the penalty coefficient within a certain range and the kernel function parameter γ in the range of [$2^{-8}$, $2^{8}$]. The optimal parameters obtained are C = 2.1534 and γ = 0.7675, and the energy consumption prediction model is constructed using these optimal parameters. (3) Based on the SVR model with optimal parameter settings, the energy enhancement index is predicted using the testing set, and the predictive performance is compared with that of linear kernel SVR, polynomial kernel SVR, sigmoid kernel function, and ARIMA model. The generalization ability of each model is evaluated using root mean square error (RMSE) and mean absolute percentage error (MAPE). The prediction performance of each model is depicted in Table 5.

**Fig. 6:**
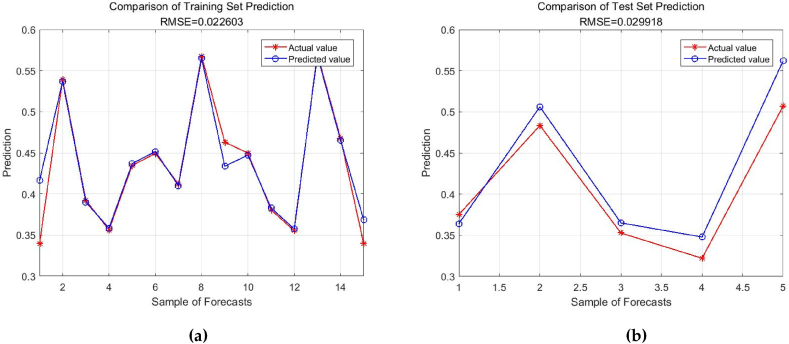
Prediction results of SSA-SVR model. (a)Test set; (b)Training set.

According to Table 5, the Gaussian kernel SVR model has the lowest root mean square error and the lowest mean absolute percentage error. It can be concluded that the Gaussian kernel SVR model exhibits superior generalization performance and its predictive effectiveness surpasses other models. Therefore, based on the optimal parameter configuration, the Gaussian kernel SVR model is employed in this study for the analysis and prediction of Chongqing's energy structure. Predicting future trends of energy structure based on the influencing factors, training and predicting data using the SSA-SVR model. The results are shown in Fig. 6 (a,b).

### 5.3 Prediction of Chongqing's energy consumption structure

In this research, the variables including green finance (GF), energy intensity (NQ), carbon emission intensity (TQ), R&D expenditure (RDJ), population scale (R), and household income level (CK, NK), that affect the energy structure in Chongqing are considered as control variables, while the remaining variables are referred to as non-control variables. The predicted values of the control variables from 2021 to 2030 are set based on the government's planned economic and social development goals, while the non-control variables are trained using the ARIMA model to predict specific data from 2021 to 2030.

#### 5.3.1 Prediction of control variables

The practical experience of achieving a well-off society in all aspects in 2020 has shown that the Chinese government can achieve the planned economic and social development goals through top-down and reasonable control. The control variables include GF, NQ, TQ, RDJ, CL, R, CK, NK.

In terms of green finance development, in November 2017, the Chongqing Branch of the People's Bank of China, in collaboration with seven other institutions, officially issued the “Chongqing Green Finance Development Plan (2017–2020)" and “Accelerating the Citywide Action Plan for Green Finance Development (2017–2018)". As the first green finance “development plan” and “action plan” in Chongqing, it aims to comprehensively and systematically promote green finance work in Chongqing, and to realize the development of a green finance system with a diverse organizational structure, abundant product variety, improved infrastructure, strong policy support, and efficient market operation by 2020. Since August 2019, when the green finance reform and innovation pilot zone was finally approved, Chongqing's green development has entered a new chapter. The balance of green loans in Chongqing increased by 40.2 % compared to the previous year, and the proportion of green loans to total loans was nearly 10 %, with a year-on-year growth rate of 4.5 %. The balance of green bonds in Chongqing is 2.7 times that of the beginning of the year. It supports the issuance of innovative green bond products such as small and medium-sized enterprise green finance collective bonds, climate bonds, blue bonds, and green asset-backed securities (ABS) for enterprises and projects, and supports corporate financial institutions in issuing green finance bonds. The balance of green bonds is striving to exceed 30 % of the city's total at the end of the “14th Five-Year Plan” period. Based on the above, the average annual growth rate of the comprehensive green finance index is determined to be 4.5 %.

According to the “14th Five-Year Plan and 2035 Vision Outline for National Economic and Social Development of Chongqing” issued in December 2020, during the “13th Five-Year Plan” period, the annual average growth rate of per capita disposable income of residents was 8.9 %. It is expected that during the “14th Five-Year Plan” period, the annual average growth rate of per capita disposable income of residents will be maintained at 6.5 %.

In terms of energy intensity, during the “13th Five-Year Plan” period, the annual average decrease rate was 3.72 %. In terms of carbon emission intensity, during the “13th Five-Year Plan” period, the annual average decrease rate was 3.9 %. The national “14th Five-Year Plan” for the modern energy system requires a cumulative reduction of 18 % in CO2 emissions per unit of GDP from 2021 to 2025. Based on this, the annual average decrease rate of carbon emission intensity in Chongqing is set at 3.6 %. The cumulative reduction of unit GDP energy consumption in Chongqing is set at 13.5 % from 2021 to 2025, resulting in an annual average decrease rate of 2.7 % in unit energy consumption. According to the United Nations' 2030 Sustainable Development Goals set in 2015, the global average annual decrease rate of energy intensity is required to be 2.6 %, thus determining the annual average decrease rate of energy intensity in Chongqing to be 2.6 %. In terms of R&D expenditure, the “14th Five-Year Plan” period requires a total increase rate of 0.4 % over five years. This research project sets the annual average growth rate of R&D expenditure in Chongqing at 0.08 %. The “Chongqing Population Development Plan (2016–2030)" expects the permanent population and registered population in Chongqing to reach around 36 million by 2030. The permanent population of Chongqing in 2021 was 34.1466 million, with an average population growth rate of 0.6 %.

Based on the government's formulated economic and social development goals, the predicted values of the seven variables, including GF, NQ, TQ, RDJ, CL, R, CK, NK for the period 2021–2030, are calculated and presented in Table 6.

The non-regulated variables include energy prices (N), industrial structure (C), household consumption structure (CE), the proportion of energy consumption in agriculture, forestry, animal husbandry, and fishery (NX), the proportion of energy consumption in daily life (SX), government-funded R&D expenditure (K), number of patent authorizations (ZS), degree of marketization (S), and environmental regulations (ER).

Considering that all variables are non-stationary time series and the factors causing non-stationarity have certain randomness, this study uses the ARIMA model to predict non-regulated variables. Specifically, N and K are modeled with ARIMA (1,0,0), CE, NX and ER are modeled with ARIMA (0,1,0), K is modeled with ARIMA (0,2,1), ZS is modeled with ARIMA (1,1,0), and the S is modeled with ARIMA (0,1,1). The predicted values of various influencing variables on energy structure are derived through calculation and displayed in Table 7.

**Table 7:** The predicted values of influencing variables on energy structure.

Year	N	C	CE	NX	SX	K	ZS	S	ER
2021	99.48	1.27	32.57	0.01	0.10	896125.80	54490.84	9.35	0.11
2022	99.62	1.21	32.57	0.01	0.10	963529.60	58497.54	9.56	0.09
2023	100.00	1.15	32.57	0.01	0.09	1030933.40	60572.52	9.77	0.08
2024	100.40	1.11	32.57	0.01	0.09	1098337.10	63410.15	9.97	0.07
2025	100.70	1.07	32.57	0.01	0.09	1165740.90	65946.68	10.18	0.05
2026	100.87	1.05	32.57	0.01	0.08	1233144.70	68602.09	10.39	0.04
2027	100.93	1.04	32.57	0.01	0.08	1300548.50	71210.56	10.60	0.02
2028	100.92	1.03	32.57	0.01	0.09	1367952.30	73837.57	10.81	0.01
2029	100.87	1.02	32.57	0.01	0.09	1435356.10	76457.25	11.02	0.00
2030	100.83	1.02	32.57	0.01	0.09	1502759.80	79079.83	11.23	0.00

We employed the ARIMA model, a time series model, to predict the energy prices in Chongqing. The results confirmed an R-squared value of 0.5386 and an average relative error of 0.0572. Due to the presence of extreme values in the energy price data, the fitting effect was not satisfactory, primarily influenced by three periods: 2008–2010, 2012–2016, and 2019–2020. In the period of 2008–2010, which encompassed two years after the financial crisis, the global energy supply chain was affected, resulting in a sharp decline in energy prices in 2009. In the period of 2012–2016, the global economy experienced a sustained downturn, and China was undergoing a transition from an industrial-based economy to a service-based economy. Moreover, countries around the world were shifting their focus on energy structure towards low-carbon fuels, leading to sluggish growth in energy consumption. Meanwhile, the rapid development of technology provided strong support for the growth of renewable energy, causing a sharp decline in global energy prices in 2015. As a result, energy prices in Chongqing remained low for four consecutive years. In the period of 2019–2020, there was downward pressure on the global economy, coupled with the large-scale outbreak of COVID-19, leading to a decline in international energy prices. The interdependent effect caused the cost of energy extraction in China to increase, resulting in a substantial decrease in energy prices. After eliminating extreme values, the energy prices in Chongqing were simulated again, and the results confirmed an R-squared value of 0.6873 and an average relative error of 0.0382.

We predict of the future values for the energy structure sophistication index in Chongqing from 2021 to 2030. Building upon the predicted values of the 7 regulatory variables and 9 non-regulatory variables obtained above, this paper employs the SSA-SVR model to forecast the energy structure sophistication index in Chongqing from 2021 to 2030. The prediction results are displayed in Table 8.

**Table 8:** Prediction of the energy structure sophistication index in Chongqing (2021–2030).

Year	Predicted values	Year	Predicted values
2021	0.666909	2026	0.735082
2022	0.680544	2027	0.748717
2023	0.694179	2028	0.762352
2024	0.707813	2029	0.775986
2025	0.721448	2030	0.789621

**Fig. 7:**
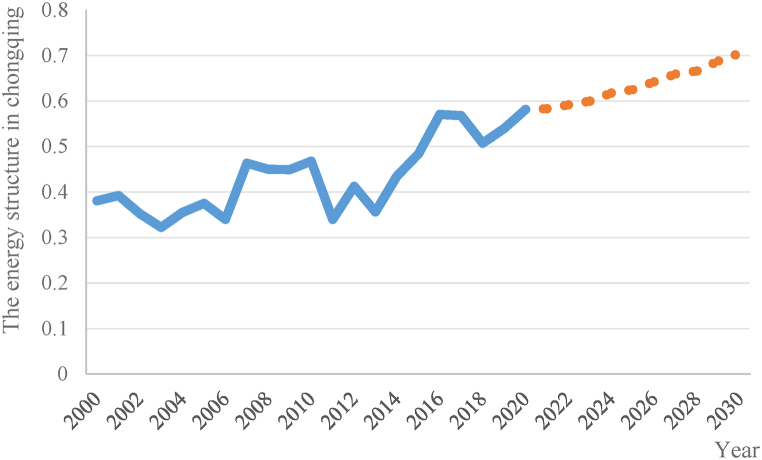
Predicted value of Chongqing's energy structure sophistication index (2021–2030).

Using the predicted data of Chongqing's energy structure starting from 2000, the forecast curve of the energy structure sophistication index is illustrated in Fig. 7. As depicted in Fig. 7., the energy structure sophistication index in Chongqing demonstrates a sustained growth trend, increasing from 0.380582 in 2000 to 0.581554 in 2020, at a growth rate of 2.64 %.

Our predictive model forecasts that the energy structure sophistication index in Chongqing will reach its peak value of 0.789621 in the year 2030, confirming that the energy structure sophistication index in Chongqing maintains a high growth trend under the promotion of green finance development, and the rate of future energy structure optimization is expected to further accelerate.

Utilizing data from the Chongqing Energy Statistical Yearbook on electricity consumption, natural gas consumption, and inter-provincial dispatch of electricity, the research project takes the value of total electricity and natural gas consumption in Chongqing as a starting point, which is 26.565 million tons of standard coal in 2021. In accordance with the “Chongqing Energy Development 14th Five-Year Plan (2021–2025)" issued by the Chongqing Municipal People's Government, considering the economic development and energy consumption in Chongqing, the annual growth rate of total electricity and natural gas consumption is set at 5 %. The specified data is presented in Table 9.

**Table 9:** Prediction of the proportion of coal energy consumption in Chongqing. (ten thousand tons of standard coal).

Year	Set value of natural gas and electricity consumption	Predicted value of coal consumption proportion	Year	Set value of natural gas and electricity consumption	Predicted value of coal consumption proportion
2021	2656.5	43.50 %	2026	2960.318	38.25 %
2022	2717.264	42.58 %	2027	3021.082	37.50 %
2023	2778.027	41.70 %	2028	3081.846	35.78 %
2024	2838.791	40.85 %	2029	3142.609	34.08 %
2025	2899.555	40.03 %	2030	3203.373	33.41 %

Based on the predicted data of the energy structure sophistication index in Table 8., the forecast of the coal energy consumption proportion in Chongqing is conducted.

Table 9 illustrates that the proportion of coal consumption in Chongqing is projected to decrease by nearly 20% points between 2021 and 2030. The forecast results indicate a declining trend in coal consumption in Chongqing with an accelerating rate of decline.

The research results ensure the feasibility of Chongqing's “14th Five-Year Plan” for energy and the 2030 peak carbon emissions target, and the comprehensive analysis leads to the following three conclusions. First, the data shows that the proportion of non-fossil energy consumption in Chongqing was close to 20.9 % in 2020. Based on calculations, in 2025, the development of green finance is expected to maintain a growth rate of 4.5 %, with coal consumption accounting for approximately 40.03 % in Chongqing, while natural gas consumption is estimated to be around 21.91 %, non-fossil energy consumption accounts for 27 %, and the proportion of oil consumption is 11.06 %.The forecast results indicate that Chongqing is expected to achieve the energy planning goals set by the Chongqing Municipal People's Government in the “14th Five-Year Plan for Energy Conservation and Emission Reduction” in 2025, with coal consumption accounting for around 40 % of the total energy consumption, petroleum consumption maintaining 15 %, natural gas consumption reaching 20 %, and non-fossil energy accounting for 25 %. Second, when the level of green finance in Chongqing is improved according to the “14th Five-Year Plan,” the proportion of coal consumption will continue to decrease and maintain a relatively stable ratio, enabling Chongqing to achieve the carbon peak target before 2030. Third, in 2025, Chongqing can achieve the energy planning goals of the “14th Five-Year Plan” while maintaining green development and economic growth, with a 14 % reduction in energy consumption per unit of regional GDP compared to 2020.

### 5.4 Robustness assessment

To assess the predictive performance of the SSA-MFD-SVR-ARIMA models, three models, including SSA-LSTM, LSTM for time series prediction using sparrow algorithm, and the grey prediction model, were chosen to forecast energy structure, validating the reliability and truthfulness of the research outcomes.

#### 5.4.1 Overview of LSTM and GM(1,1) models

With the advancement of artificial intelligence, the Long Short-Term Memory Artificial Neural Network (LSTM) model has found extensive applications in time series prediction within the financial field. The LSTM model is a deep learning model that supports time series and can fit convergent nonlinear and chaotic data in a time series manner. It can also perform regression prediction on multiple input and output variables. Additionally, in the realm of time series prediction models, the grey dynamic model GM(1,1) has been extensively utilized for forecasting energy consumption [83]. It is a time response function for a first-order differential equation of a variable sequence, utilized for quantitative analysis and prediction purposes. Nevertheless, both LSTM and grey system prediction models still confront typical challenges in time series prediction models, including low prediction accuracy and weak generalization capabilities. Validation is needed for both prediction accuracy and precision.

#### 5.4.2 Results of SSA-LSTM prediction

**Fig. 8:**
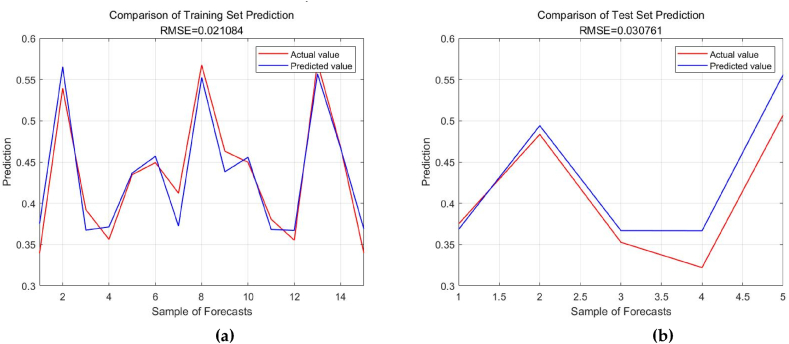
Prediction results of SSA-LSTM model. (a)Test set; (b)Training set.

In accordance with the aforementioned method and the same division of input variables and training-testing sets as in the preceding section, SSA-LSTM regression models and LSTM time series models were developed for forecasting energy consumption structure. A deep learning framework was employed to construct the LSTM network, and it was trained using training dataset. Subsequently, the trained LSTM model was applied for regression analysis, the trained LSTM model was and the fitting results on the test data are depicted in Fig. 8(a and b).

The root mean square errors (RMSE) of the training and testing sets are 0.0211 and 0.0308, respectively. The model fits well with small prediction errors. However, since the RMSE of SSA-LSTM is higher than that of SSA-SVR, its fitting performance is inferior to SSA-SVR. Consequently, we employ the SSA-LSTM model for further predictions to validate whether it can facilitate energy structure optimization under the backdrop of green financial development in Chongqing.

#### 5.4.3 Results of LSTM time series

**Fig. 9:**
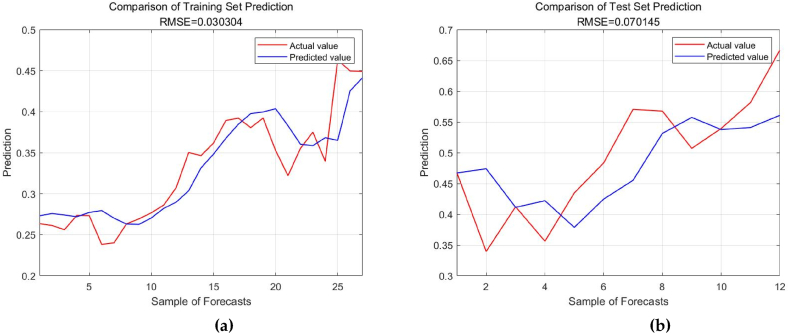
Prediction results of LSTM model. (a)Test set; (b)Training set.

Upon verification, we observed that most of the time series predictions using historical data from 2000 to 2020 exhibited signs of overfitting. This is attributed to the limited sample size, which impacted prediction accuracy and fit. To address this, we employed historical data of energy sophistication index from 1980 to 2020 for time series predictions. The data from 1980 to 2007 were designated as the training set, whereas the years 2008–2020 constituted the testing set. Fig. 9(a and b) depict the fitting results of the training and testing sets. From the figures, it is evident that the RMSE for the training and testing sets is 0.0303 and 0.0701, respectively. The fitting performance is noticeably inferior to the regression prediction models.

#### 5.4.4 Results of grey system prediction

**Fig. 10:**
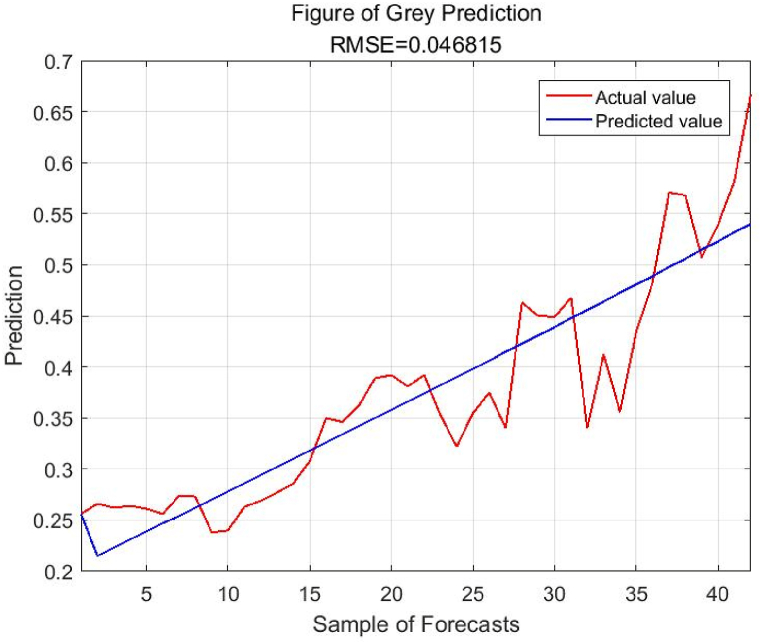
Prediction results of grey system model.

The GM(1,1) model generates an accumulated sequence with pronounced exponential regularity by cumulatively summing a discrete random original sequence. Subsequently, after parameter estimation through sequence modeling, the calculated values are cumulatively subtracted to derive the predicted values. The establishment of a first-order, single-variable grey system model (GM(1,1) model) encompasses five steps: grey sequence generation, examination of exponential regularity, parameter estimation for model, determination of prediction model, model error verification, and grey prediction. Fig. 10 below displays the historical data and forecasted results of Chongqing's energy complexity index from 1980 to 2020. The GM(1,1) prediction model exhibits an average relative error (MRE) of 9.98 %, with a fitting accuracy of 90.02 % in relation to the fit values compared to the actual values, and it has successfully passed the residual error test.

#### 5.4.5 Comparison of prediction results

We employed three methods, SSA-LSTM regression prediction, LSTM time series prediction, and grey prediction model, to validate the previous research findings. This simultaneously confirmed the advantages of the SSA-MFD-SVR-ARIMA model in predicting Chongqing's energy structure. To compare the generalization abilities of the models, we employed evaluation criteria such as root mean square error (RMSE), mean absolute percentage error (MAPE), coefficient of determination (R2), mean absolute error (MAE), and mean bias error (MBE). The prediction results of each model are presented in Table 10.

**Table 10:** Deviation analysis of test set.

	RMSE	R^2^	MAE	MBE	MAPE
SSA-SVR	0.0300	0.8345	0.0254	0.0210	5.6151
SSA-LSTM	0.0308	0.8250	0.0250	0.0223	7.3342
LSTM	0.0701	0.6573	0.0556	0.0458	14.4071
GM(1,1)	0.0468	0.8092	0.0365	0.0952	9.6848

**Fig. 11:**
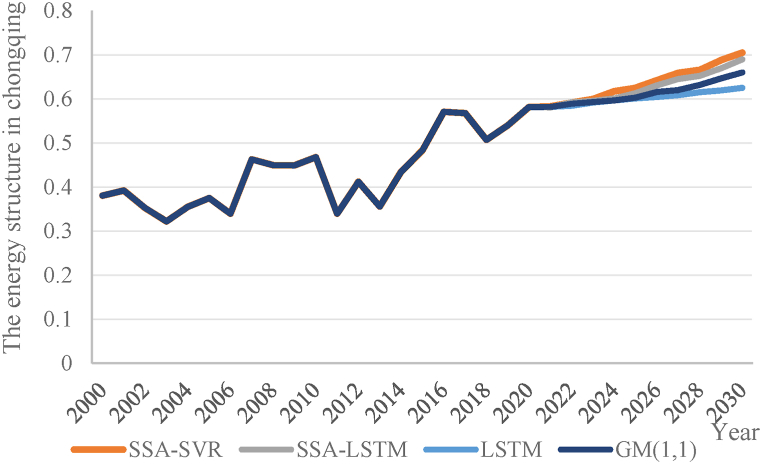
Prediction results of four models.

(1) Among the four models, the testing set RMSE, MAE, MBE, and MAPE of the SSA-SVR model are all lower than the other three models. This verifies that the SSA-SVR model has the best fitting performance and the most robust explanatory capabilities. SSA-LSTM regression prediction exhibits a superior fitting degree compared to time series prediction. (2) In Table 10, the RMSE values for the GM(1,1) model, LSTM time series forecasting model, and SSA-LSTM regression forecasting model are 0.0468, 0.0701, and 0.0308, respectively. Notably, the SSA-LSTM model exhibits the lowest RMSE value. This affirms that the regression prediction model better evaluates the developmental trends in non-linear energy structure data. Meanwhile, the prediction accuracy of the GM(1,1) model outperforms that of the LSTM time series prediction model. This is attributed to the GM(1,1) model's suitability for small samples, while the LSTM time series model is better suited for datasets with large samples and pronounced data volatility. The forecasted outcomes of Chongqing's energy sophistication index for the period 2000 to 2020 b y the four models are depicted in the accompanying Fig. 11.

Utilizing the previously established values for natural gas and electricity consumption in Chongqing, in conjunction with predictions made by the four models for the energy sophistication index from 2021 to 2030, we calculated the proportion of coal consumption in Chongqing (as presented in Table 11).

**Table 11:** Predicted value of coal consumption proportion.

Year	SSA-SVR	SSA-LSTM	LSTM	GM(1,1)
2021	43.50 %	43.03 %	43.86 %	43.47 %
2022	42.58 %	41.92 %	43.29 %	42.60 %
2023	41.70 %	40.79 %	42.35 %	41.84 %
2024	40.85 %	40.42 %	41.82 %	41.08 %
2025	40.03 %	39.89 %	41.53 %	40.71 %
2026	38.25 %	38.70 %	40.81 %	40.32 %
2027	37.50 %	36.90 %	40.32 %	39.69 %
2028	35.78 %	35.47 %	39.89 %	38.42 %
2029	34.08 %	34.78 %	39.56 %	37.98 %
2030	33.41 %	33.06 %	39.13 %	36.86 %

As indicated in Tables 11 and in accordance with the SSA-SVR and SSA-LSTM regression prediction models, the proportion of coal consumption in Chongqing is expected to drop below 40 % in 2025 under the influence of green financial development. SSA-LSTM confirms the credibility of the previous research results. In the LSTM and GM(1,1) time series prediction models, the proportion of coal consumption in Chongqing is projected to remain above 40 % in 2025. We attribute this outcome to the intricate interplay of internal and external influencing factors within the energy system. Time series prediction models tend to overlook the driving impact of factors affecting energy structure, and their consideration of temporal data correlations is inadequate. Nevertheless, due to the uncertainty of regarding factors affecting energy structure and the limited number of analyzable samples, further enhancements are required for regression prediction models.

Our observations indicate that the SSA-MFD-SVR-ARIMA model outperforms the SSA-LSTM regression prediction model, LSTM time series prediction, and the grey prediction model in terms of accuracy and stability when forecasting energy structure. Furthermore, the findings from the SSA-LSTM regression prediction model and LSTM time series prediction support the assertion that, with the support of green finance, Chongqing can attain its goal of reducing coal consumption to below 40 % by 2025.

## 6 Path analysis

Chongqing offers a green financial approach to optimize its energy structure. The four pathways of green finance, through green equity investment, energy digital finance, environmental equity financing, and industrial development fund, are dedicated to promote green innovation in businesses and optimize the entirety of energy consumption structure in the low-carbon industry sector.

**Fig. 12:**
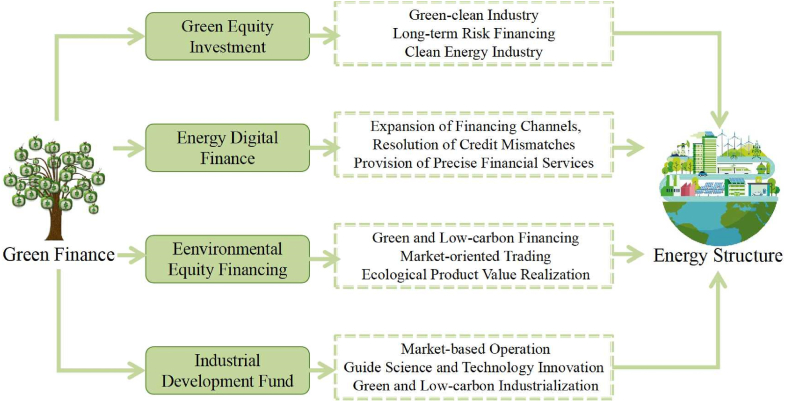
The path of the optimization of energy structure through green finance in Chongqing. (For interpretation of the references to colour in this figure legend, the reader is referred to the Web version of this article.)

The routes of the optimized energy structure of green finance in Chongqing is presented in Fig. 12.

### 6.1 Green equity investment

As of 2020, green credit and green securities accounted for approximately 98 %, while green equity financing only accounted for 2 %. The development of green equity investment in China is not yet mature and requires further improvement. Responding to this, the Shanghai Stock Exchange's 2021 Social Responsibility Report called for the optimization of green equity financing services and increased funding for the green low-carbon industry sector. Chongqing followed suit, releasing the “14th Five-Year Plan” Implementation Plan for “Waste-Free Cities” (draft for soliciting opinions) in October 2022, proposing to “guide social capital to participate in green investment in the form of government and social capital cooperation (PPP) or equity investment.” The main funding from green equity investment will be directed towards green energy consumption industries that meet the demand for clean energy and support long-term risk financing.

The green equity investment mainly channels funds into the green energy industry, meeting the demands of clean energy and cooperating with long-term risk financing.

(1) The funds flow into the green industry. This green industry encompasses environmental protection, green technology, ecological agriculture, and green service sectors. The majority of enterprises within these fields employ environmentally conscious practices such as utilizing green technologies, clean energy, and developing green products. Additionally, they operate under a sustainable and green business model. Investors who purchase equity in green enterprises gain decision-making powers and production control while supporting sustainable operations. Moreover, investing in environmentally friendly businesses effectively helps reduce operating risks by diversifying portfolios. Furthermore, green enterprises utilize energy-saving, resource integration, and clean production technologies to optimize energy consumption structures while minimizing fossil fuel consumption and resource utilization.
(2) Cooperate with long-term risk financing. Small and medium-sized green, low-carbon, and technological enterprises, as investment targets, possess long investment recovery periods, high technological uncertainty levels, and significant project risks. It is proposed to establish a financial service model primarily dominated by private equity and venture capital. This model is used to encourage venture capital funds to incubate green, low-carbon technology enterprises, increase investments in critical renewable energy technologies, and achieve the conversion of enterprise scientific and technological projects and achievements. Concurrently, support equity investment funds in carrying out green projects or enterprise mergers and acquisitions, and guide private equity investment funds to cooperate with regional equity markets to provide the necessary conditions for the transfer listing of green assets (enterprises). Green transformation, technological innovation, and the acceleration of renewable energy development and storage can reduce the consumption of fossil fuels and achieve regional energy structure adjustments.
(3) Meet the demands of clean energy industry. Equity investment in upstream and downstream enterprises of the resource integrated utilization industry and the renewable energy industry chain can ensure the safety of enterprise capital chains, promote original new energy technology innovation, and establish clean energy production bases and industrial parks. In green industrial parks, enterprises can coordinate clean energy supply and demand and build a circular economy that saves resources, reduces traditional energy consumption, and accelerates energy system transformation and energy structure adjustment.

### 6.2 Energy digital finance

The uniqueness of digital finance in the energy sector lies in its utilization of cutting-edge technologies such as artificial intelligence and big data, which provide unprecedented financing opportunities for emerging energy industries and expedite the transformation of traditional energy sources. The path towards optimized energy structures through digital finance lies in the expansion of financing channels, resolution of credit mismatches, and the provision of precise financial services.

(1) Expand financing channels. Digital finance in the energy industry leverages technologies such as big data, blockchain, artificial intelligence, and others to integrate traditional finance with emerging technologies, thereby creating an information-sharing platform that supports the energy sector. By consolidating fragmented funds in the financial market, reducing transaction costs, and lowering barriers to financial services for the energy industry, digital finance improves the availability of green funds and provides funding security for traditional and new energy sectors' green projects and technology research and development. Moreover, digital finance accurately assesses risks associated with corporate green projects and quickly and precisely addresses any green funding gaps through integrated information. In this way, it accelerates the supply of clean energy, leading to adjustments in the energy structure.
(2) Address credit mismatches. Digital finance in the energy industry relies on information technology to efficiently integrate and match enterprise information using tools such as big data and algorithms. This setup facilitates the establishment of a digital credit system and a green project assessment platform, thereby alleviating the adverse effects of information asymmetry, resulting in moral hazard and unduly selective subjectivity, effectively solving the credit mismatch issues of green projects in the energy industry and contributing to promote better energy structures.
(3) Precision financial services. The digital platform simplifies the financial service process and improves financing efficiency, allowing clean energy companies to acquire necessary funds in a timely manner. Digital finance also provides individualized financial services that match clean energy project financing demands.

In summary, digital finance in the energy industry alleviates financing constraints, guiding more financial and human resources towards energy technology research and development, promoting innovation, and driving efficient energy utilization. These efforts ultimately lead to an optimized energy structure.

### 6.3 Environmental equity financing

The municipality of Chongqing persistently develops novel environmental equity financing tools to provide crucial support for promoting resource recycling and carbon emission reduction. Since 2014, the municipality has successively established the Carbon Emission Trading Center, Pollution Emission Trading Center, and Forest Rights Trading Center, and the environmental equity trading market is gradually taking shape. In the environmental equity field, the first mortgage loan business for pollution emission rights landed in Chongqing in October 2015, the first carbon quota pledge financing business landed in Chongqing in April 2017, and the first ‘carbon hook’ loan business landed in Chongqing in June 2022. The environmental equity financing market has become an indispensable domain in Chongqing's future energy conservation and carbon reduction process.

Green finance utilizes environmental equity financing tools to promote energy structural optimization. It mainly operates through two ways of direct financing and indirect financing, viewed from both the financing inflow and outflow perspectives, and analyzes the adjustment of energy structure driven by environmental equity financing from three angles of expanding green and low-carbon financing channels, improving market-oriented trading mechanisms, and improving ecological product value realization mechanisms.

Improve market-based trading mechanisms. Environmental equity tools operate through direct financing mode in carbon emission trading, carbon quota and CCER markets, providing standard guidance for private capital to participate in high-quality ecological environment projects and effectively mobilizing private investment enthusiasm. Before business transactions, companies need to weigh innovative emission reduction costs and equity divestment costs to determine the transaction direction. Equity income parties and capital outflow parties are mainly high-polluting enterprises that pay for the pollutants emitted beyond the limit to reduce emissions. Equity selling parties and capital inflow parties are mostly clean enterprises that undertake ecological environment projects and pollution manufacturing industries that meet emission reduction standards. These enterprises obtain funds from the market, ensure the upgrading of industry, and reduce carbon emissions under the consumption of fossil energy, thereby adjusting the overall energy structure.

Expand channels for green and low-carbon financing. Indirect tools for environmental equity financing create characteristic products such as carbon emission quotas, wetland carbon sinks, pollutant discharge rights, and forestry carbon sinks as collateral for traditional financing tools to carry out financial services such as mortgage loans for pollutant discharge rights and pledge financing for carbon quotas. The aim is to activate market funds, provide green project financing funds for capital inflow parties, guide capital towards the green low-carbon field, increase the proportion of non-fossil energy consumption, and achieve a rational energy consumption structure.

Improve the mechanism for realizing the value of ecological products. Clean and green enterprises, as both equity lenders and funding recipients, are able to bring financial resources into the market and encourage the development of energy-saving and carbon-reducing technologies, facilitating the use of fossil energies in a cleaner and more low-carbon way and promoting the replacement of traditional energy sources with clean energies. At the same time, based on the actual situation and needs of local green development, they can improve the comprehensive industrial chain of ecological products, assist in the green transformation of traditional industries, promote the healthy development of the green industry and promote the adjustment of energy structure at the industrial level.

### 6.4 Industrial development fund

The Local Government of Chongqing has been consistently increasing its support for green industries by seizing investment opportunities, resulting in rapid development for green industry funds. The Chongqing Government and social capital have jointly established a regional green development fund, which, in collaboration with banking and financial institutions, carries out loan-linked financing to empower sustainable economic growth in green industries. At the same time, the Chongqing Government is exploring green industry guarantee funds, while focusing on the new energy domain by providing financial support for technological breakthroughs in the industry and clearly delineating the investment direction of “dual-carbon” investments.

Green finance optimizes energy structures through its industrial development funds, which involve green private equity funds, zero-carbon risk investment funds, and carbon neutrality specialty funds, fostering financing for green and low-carbon clean environmental projects. By establishing a sound market operation mechanism, actively utilizing technological innovation funds, and accelerating the industrialization of green and low-carbon development, the inner workings and operative paths of green industry development funds are analyzed, providing targeted development directions for green finance in Chongqing and expediting the ultimate goal of energy structure adjustment.

Establish and improve market-based operation mechanisms. The industrial development fund follows a market-based approach, providing green financing channels for environmentally-friendly enterprises through open issuances. The standardized market operations regulate the scale of funding, production capabilities, and technological strength of low-carbon industries, allowing investors to conveniently purchase high-quality industrial funds in the public market. A large number of green and low-carbon small and medium-sized enterprises will no longer be limited by traditional financing models that result in funding shortages. Simultaneously, the fund injects new momentum into enterprise development, catalyzing regional energy transformation and environmental sustainability.

Actively utilize funds to guide science and technology innovation. The industrial fund focuses on the transformation of green patents and investment in low-carbon technologies, guiding capital towards low-carbon and environmentally-friendly industries that serve the technological innovation needs of low-carbon enterprises. In conjunction with a diversified, multi-level, and multi-channel technological investment and financing system, the fund injects powerful energy into regional green and sustainable development and provides an important safeguard for the utilization and consumption of renewable energy in the region.

Accelerate the process of green and low-carbon industrialization. The industrial development fund guides investors to pay attention to new energy, energy conservation and environmental protection industries, and low-carbon development industries, promoting the expansion of clean and low-carbon industries. In turn, this encourages the healthy development and optimization upgrading of industrial industries, achieving the low-carbon transformation of industrial industries and ultimately optimizing the energy structure.

## 7 Conclusion and countermeasures

This investigation examines how green finance can optimize Chongqing's energy structure from 2000 to 2020. The dependencies and contribution variables are quantitatively calculated utilizing the Copula function. The SSA-MFD-SVR-ARIMA model is applied to predict the energy structure under sustainable development of green finance from 2021 to 2030, which provides uniquely insights for adjusting the energy structure via green finance. The predictive estimation highlights some interesting and eye-catching conclusions. The main conclusions of this research are as follows: (1) From 26 variables, 16 highly dependent variables that significantly affect Chongqing's energy structure are selected based on the Copula function, including green finance, energy prices, residents' income levels, industrial structure, residents' consumption structure, energy consumption ratio of agriculture, forestry, animal husbandry and fishery, energy intensity, carbon emission intensity, R&D expenditure, government spending on scientific research funds, granted patents, number of scientific and technological personnel, population, urbanization level, degree of marketization, and environmental regulations. Among them, the dependence between green finance and energy structure is 10.80 %, with Granger causality. The contribution of green finance cannot be ignored in the optimization of energy structure. (2) The mechanism of how green finance optimizes the energy structure includes industrial upgrading, technological innovation, market financing, and policy guidance. The development of green finance in Chongqing is in line with the aforementioned channels of transmission. (3) Predictive findings demonstrate that the energy structure sophistication index in Chongqing has shown sustained growth from 0.380582 in 2000 to 0.581554 in 2020, at a growth rate of 2.64 %, and it is expected to reach its peak value of 0.789621 in 2030 under the promotion of green finance development. By 2025, coal consumption is expected to account for about 40.03 % under the development of green finance, maintaining a growth rate of 4.5 %, while non-fossil energy consumption accounts for 27 %. These predictions ensure the feasibility of Chongqing's “14th Five-Year Plan” energy plan. (4) Based on predictive estimation, a path for optimizing Chongqing's energy structure from the perspective of green finance is proposed, which consists of green equity investment, energy digital finance, environmental equity financing, and industrial development fund.

The four novel routes for Chongqing to optimize its energy structure through green finance. Relying on the above findings, the four guarantee strategies for pathways are elaborated as follows.

(1) Financing Mechanisms

Firstly, harnessing the leadership role of financial institutions. Chongqing boasts an extensive network of rivers, harboring significant hydroelectric development potential. Capitalizing on hydropower resources, nurturing innovative green financial developments in Chongqing, establishing dedicated green credit institutions, and gradually shaping a green “funding oasis” in western China. The Fengdu County wind power project in Chongqing, deemed a valuable asset by banks, not only generates profits but also drives clean energy development. Driving the expansion and augmentation of green finance, directing institutions to prioritize high-quality green projects while enhancing credit processes. Secondly, innovating new types of green investment and financing tools. Chongqing's financial industry is vibrant, accounting for 8.8 % of the total economic output in 2021 and becoming a pivotal industry driving economic development. Consequently, Chongqing's Liangjiang New Area, as a national-level pilot industrial park, is in the process of exploring a pathway for climate-centered investment and financing development. Harnessing the specialized financing functions of finance, founding China's inaugural Climate Investment and Financing Promotion Center, and constructing an all-encompassing climate investment and financing service platform. Relying on the construction of climate investment and financing project and enterprise databases in industrial pilot zones and engaging in international cooperation. Tracing back to the origins, undertaking climate governance from the vantage point of energy consumption and supply, introducing specialized financing options like energy efficiency credits and contract energy management to support the emerging energy sector with financing services. Chongqing commenced its carbon emissions trading platform in 2014, and the market trading system is now fundamentally sound. Building upon this foundation, there is an immediate necessity to invigorate innovation in carbon finance. Researching and implementing innovative carbon finance initiatives, including carbon futures, carbon repurchases, carbon collateral, and carbon asset management, while exploring the creation of comprehensive carbon accounts for businesses and individuals to reverse-regulate energy consumption and supply. Executing the “Green Easy Loan” refinancing and “Green Bill Exchange” rediscounting special support program to offer directed assistance to financial institutions within the jurisdiction for green credit and green bill discounting.

(2) Innovation Mechanisms

Firstly, innovating distinctive “carbon neutrality” capital market products. Chongqing's capital market, following a decade of development, has acquired sufficient the capability and efficiency to create novel green financial products and service approaches. Green debt financing instruments, climate investment and financing tools, and specific project credit products serve as new directions for financial innovation in Chongqing, furnishing reference solutions for realizing energy structure optimization objectives. For instance, the 2021 s tranche of Green Medium-Term Notes (Carbon Neutral Bonds), green accounts receivable debt financing plans, carbon emissions quota collateralized financing, wastewater discharge rights-secured loans, water use rights-secured loans, certified loans for new energy subsidies, and “carbon-linked” specialized products. Secondly, establishing a comprehensive digital connectivity platform. Chongqing has established the Western Data Trading Center, ranking ninth in digital comprehensive development, progressively nurturing novel digital models and formats. Establishing the “Yangtze Green Finance” comprehensive green financial big data service system; enhancing the institutional framework of the Chongqing “Carbon Benefit” platform. Capitalizing on innovative digital connectivity platforms powered by big data technology, engaging in financing for renewable energy projects, and offering enterprises opportunities for interconnectivity with financial institutions. Thirdly, fully harnessing the guiding and driving role of monetary policy tools. Chongqing is constructing a small-loan financial model, utilizing inclusive credit to open up financing channels for “specialized and refined” enterprises engaged in emissions reduction. Monetary tools facilitate green and low-carbon development in Chongqing, encompassing rediscounting operations, refinancing operations, carbon emission reduction support tools, and environmental rights collateralization tools. For instance, “Green Easy Loan” refinancing and “Green Bill Exchange” rediscounting tools, green bonds and green bond rediscounting operations, carbon emission reduction loans for high-energy-consuming enterprises, and loans collateralized with environmental rights for clean energy projects.

(3) Collaborative Mechanisms

First, drive tripartite collaboration between industry, enterprises, and government. Chongqing has established a resource platform for green industry development, maintaining a coordinated effort to extend the industrial value chain. Chongqing's green finance utilizes approaches like financing green energy projects, digital financial platforms, industrial development funds, and individual green retail products to drive interconnectivity between industry and supply chains. As an illustration, the Chongqing Green Development Fund operates in a financing-capital-social capital partnership (PPP) model, employing a three-party interconnected approach and operates in the structure of “parent fund + direct investment + carbon fund.” Secondly, the mechanism of interconnection between the financial industry and enterprises. Based on Chongqing's advantageous position as the southwestern center, it clearly defines the financial support and leadership for the Yangtze River's upper and middle reaches, which is conducive to addressing the “island effect” between banks and enterprises, and endeavoring to construct a high-level interconnected and shared green financial ecosystem. Developing a green supply chain and industrial chain financing model that integrates capital flow, logistics, and information flow into one cohesive system, establishing a closed-loop risk management framework to ensure manageable financing risks, thus steadily advancing energy structure optimization and adjustment. Thirdly, financial direction for the coordinated development of diverse sectors. Chongqing adopts small loan funds as a financial model, cultivating the mutual integration of the financial sector with manufacturing, organically evolving into a composite industrial chain system, driving synergistic development across industries. Establishing enhanced communication, coordination, and feedback mechanisms, collectively devising long-term regional goals for green and low-carbon development. Prioritizing balanced development in areas like green growth, precise poverty alleviation, pollution prevention and control, rural development, and support for small and micro-enterprises, expediting industrial convergence.

(4) Security mechanisms.

Firstly, real-time monitoring of financial risks. Chongqing's financial risk prevention model revolves around specific exchange strategies, endorsing equity trading venues, carbon emissions trading markets, and oil and gas futures trading markets, advancing the rectification of key sectors. Regulatory authorities in the financial sector should conduct real-time risk monitoring for green investment and financing risks, ensuring the implementation of systems for corporate environmental risk assessment and information disclosure. Utilizing financial technology, establish a specialized green finance risk control system, create a platform for monitoring corporate environmental risks, and implement an energy consumption measurement system to mitigate financial risks. Secondly, strengthen the facilitative role of fiscal and financial regulatory policies. Chongqing's objective is to establish a regional financial center in the upper Yangtze River region, intensifying financial regulation to stimulate capital vitality. The fiscal department should establish and refine a comprehensive, end-to-end, and widely applicable closed-loop mechanism for green finance incentives, promulgating comprehensive, integrated green finance incentive policies encompassing measures such as credit interest subsidies, bond interest subsidies, listing incentives, insurance premium subsidies, guarantee incentives, and risk-sharing. Implementing complementary measures to support green finance reform and innovation, offering financial subsidies to enterprises for green finance activities like green loans, green bonds, and green insurance, and providing risk compensation for losses in green finance activities. Thirdly, attract talent with expertise in both green finance and new energy. Research institutions like the Western Financial Research Institute, Jiangbei Finance and Economics Think Tank, and financial universities are providing Chongqing with a substantial number of high-quality interdisciplinary talents. Incentives are offered to current specialized talent in green finance and new energy as well as newly recruited talent from outside, with concurrent encouragement and guidance for financial institutions to attract and cultivate professionals in the field of green finance. Leveraging think tank research organizations to consolidate talent and intellectual resources, aiding Chongqing's green finance decision-making.

## Funding

This research was funded by 10.13039/501100007957Chongqing Municipal Education Commission A Graduate Research and Innovation Project “Mitigation Mechanism of Green Finance for the Energy Triple Dilemma in the Yangtze River Economic Belt”, grant number CYS23559. 10.13039/501100007957Chongqing Municipal Education Commission A Graduate Research and Innovation Project “Research on the Pathway of Green Finance to Enhance the Carbon Emission Reduction Potential in the Yangtze River Economic Belt under the Dual Carbon Goals”, grant number CYS23560. 10.13039/501100004500Chongqing Technology and Business University Yangtze Upper Reaches Economic Research Center Project: “Research on Carbon Peak Paths in the Chengdu-Chongqing Urban Agglomeration”, grant number KFJJ2022045. Chongqing Social Science Planning Project: “Research on Pricing Strategies for Carbon Emission Trading in Chongqing” (2019WT52). Key Humanities and Social Sciences Research Base Project of Chongqing Municipal Education Commission: “Prediction of Carbon Peak and Differentiated Emission Reduction Paths in the Chengdu-Chongqing Urban Agglomeration under the ‘Dual Carbon’ Goals” (23SKJD094).

## Data availability statement

The research-related data were not deposited in a publicly available repository. Data is contained within the article. The original data is obtained from the National Bureau of Statistics website, China Energy Statistical Yearbook, the Chongqing Municipal Bureau of Statistics website and the WIND database.

## Disclaimer/Publisher's note

The statements, opinions and data contained in all publications are solely those of the individual author(s) and contributor(s) and not of MDPI and/or the editor(s). MDPI and/or the editor(s) disclaim responsibility for any injury to people or property resulting from any ideas, methods, instructions or products referred to in the content.

## CRediT authorship contribution statement

**Sheng Zeng:** Methodology, Conceptualization. **Yangchen Yu:** Writing – review & editing, Writing – original draft, Visualization, Validation, Supervision, Software, Resources, Project administration, Investigation, Funding acquisition, Formal analysis, Data curation. **Wenze Li:** Validation, Data curation.

## Declaration of competing interest

The authors declare the following financial interests/personal relationships which may be considered as potential competing interests:Yangchen Yu reports financial support was provided by 10.13039/501100007957Chongqing Municipal Education Commission. If there are other authors, they declare that they have no known competing financial interests or personal relationships that could have appeared to influence the work reported in this paper.

## Figures and Tables

**Table A1:** The descriptive statistics of the variables.

Variable	Sample Size	Mean	Minimum Value	Maximum Value	Standard Deviation
NY	21	0.4352	0.3221	0.5816	0.0809
G	21	10393.4291	1822.0600	25002.7900	7586.7981
GZ	21	11.4381	3.9000	17.2000	3.3925
Z	21	8567.6820	655.8100	20494.3900	6995.3995
N	21	101.3476	84.5000	112.9000	6.5491
RG	21	34492.0500	6383.0000	78173.0000	23827.1730
GF	21	0.1703	0.0784	0.4793	0.0865
CK	21	19145.4300	6152.0000	40006.0000	10747.3310
NK	21	6965.9520	1900.0000	16361.0000	4646.6284
C	21	34.9139	31.1613	41.6000	2.8326
CE	21	44.5069	34.8884	53.9000	6.3991
NE	21	0.9780	0.6538	1.3223	0.1895
NX	21	0.0360	0.0104	0.0754	0.0209
GX	21	0.5325	0.4681	0.7255	0.0617
ZX	21	0.2127	0.1663	0.3273	0.0358
SX	21	0.0898	0.0622	0.1258	0.0185
LS	21	4.6377	1.0969	9.6273	2.8084
NQ	21	0.8026	0.4724	1.1356	0.2228
TQ	21	1.9146	0.7905	3.3257	0.7386
RDJ	21	1625767.0000	101294.0000	5267900.0000	1592905.2000
RDG	21	0.0171	0.0056	0.0326	0.0075
K	21	294602.0000	41089.0000	828722.0000	254073.7700
ZS	21	18788.7100	1158.0000	55377.0000	17444.4120
R	21	3276.6620	3091.0900	3416.2900	112.4069
CL	21	53.1667	35.6000	69.5000	10.1498
S	21	7.9352	4.9000	9.5000	1.3030
ER	21	0.2006	0.0886	0.4000	0.0896

**Table A2:** The results of the stationarity test for each indicator.

Variable	ADF Statistic	1 % Critical Value	5 % Critical Value	10 % Critical Value	Conclusion
NY	−9.615	−3.75	−3	−2.63	Stationarity
G	−4.957	−3.75	−3	−2.63	Stationarity
GZ	−7.466	−3.75	−3	−2.63	Stationarity
GF	−5.228	−3.75	−3	−2.63	Stationarity
Z	−5.786	−3.75	−3	−2.63	Stationarity
N	−7.996	−3.75	−3	−2.63	Stationarity
RG	−5.226	−3.75	−3	−2.63	Stationarity
CK	−5.609	−3.75	−3	−2.63	Stationarity
NK	−5.962	−3.75	−3	−2.63	Stationarity
C	−5.706	−3.75	−3	−2.63	Stationarity
CE	−6.413	−3.75	−3	−2.63	Stationarity
NE	−8.39	−3.75	−3	−2.63	Stationarity
NX	−5.066	−3.75	−3	−2.63	Stationarity
GX	−7.264	−3.75	−3	−2.63	Stationarity
ZX	−6.76	−3.75	−3	−2.63	Stationarity
SX	−4.599	−3.75	−3	−2.63	Stationarity
LS	−4.766	−3.75	−3	−2.63	Stationarity
NQ	−4.888	−3.75	−3	−2.63	Stationarity
TQ	−6.682	−3.75	−3	−2.63	Stationarity
RDJ	−7.679	−3.75	−3	−2.63	Stationarity
RDG	−7.794	−3.75	−3	−2.63	Stationarity
K	−6.667	−3.75	−3	−2.63	Stationarity
ZS	−6.284	−3.75	−3	−2.63	Stationarity
R	−7.239	−3.75	−3	−2.63	Stationarity
CL	−6.059	−3.75	−3	−2.63	Stationarity
S	−4.745	−3.75	−3	−2.63	Stationarity
H	−8.345	−3.75	−3	−2.63	Stationarity

**Table A3:** The results of the cointegration test for each indicator.

Variable	ADF Statistic for Residuals	1 % Critical Value	5 % Critical Value	10 % Critical Value	Residual Stationarity	Conclusion
NY&G	−5.357	−3.75	−3	−2.63	Stationarity	Existence
NY&GZ	−5.129	−3.75	−3	−2.63	Stationarity	Existence
NY&GF	−5.461	−3.75	−3	−2.63	Stationarity	Existence
NY&Z	−4.778	−3.75	−3	−2.63	Stationarity	Existence
NY&N	−5.22	−3.75	−3	−2.63	Stationarity	Existence
NY&RG	−5.312	−3.75	−3	−2.63	Stationarity	Existence
NY&CK	−4.786	−3.75	−3	−2.63	Stationarity	Existence
NY&NK	−4.807	−3.75	−3	−2.63	Stationarity	Existence
NY&C	−5.373	−3.75	−3	−2.63	Stationarity	Existence
NY&CE	−5.611	−3.75	−3	−2.63	Stationarity	Existence
NY&NE	−5.456	−3.75	−3	−2.63	Stationarity	Existence
NY&NX	−4.502	−3.75	−3	−2.63	Stationarity	Existence
NY&GX	−5.197	−3.75	−3	−2.63	Stationarity	Existence
NY&ZX	−5.187	−3.75	−3	−2.63	Stationarity	Existence
NY&SX	−4.984	−3.75	−3	−2.63	Stationarity	Existence
NY&LS	−4.796	−3.75	−3	−2.63	Stationarity	Existence
NY&NQ	−5.12	−3.75	−3	−2.63	Stationarity	Existence
NY&TQ	−4.903	−3.75	−3	−2.63	Stationarity	Existence
NY&RDJ	−5.183	−3.75	−3	−2.63	Stationarity	Existence
NY&RDG	−5.443	−3.75	−3	−2.63	Stationarity	Existence
NY&K	−5.475	−3.75	−3	−2.63	Stationarity	Existence
NY&ZS	−5.52	−3.75	−3	−2.63	Stationarity	Existence
NY&R	−5.324	−3.75	−3	−2.63	Stationarity	Existence
NY&CL	−5.057	−3.75	−3	−2.63	Stationarity	Existence
NY&S	−5.832	−3.75	−3	−2.63	Stationarity	Existence
NY&H	−5.358	−3.75	−3	−2.63	Stationarity	Existence

**Table A4:** Granger Causality Test Results for Each Indicator.

H0: Hypothesis	*P*-value	Result	H0: Hypothesis	*P*-value	Result
H0: NY is not a Granger cause of G	0.5426	accept	H0: G is not a Granger cause of NY	0.5583	accept
H0: NY is not a Granger cause of GZ	0.9632	accept	H0: GZ is not a Granger cause of NY	0.4816	accept
H0: NY is not a Granger cause of GF	0.9352	accept	H0: GF is not a Granger cause of NY	0.0273	reject
H0: NY is not a Granger cause of Z	0.3708	accept	H0: Z is not a Granger cause of NY	0.979	accept
H0: NY is not a Granger cause of N	0.0298	reject	H0: N is not a Granger cause of NY	0.5583	accept
H0: NY is not a Granger cause of RG	0.5242	accept	H0: RG is not a Granger cause of NY	0.5876	accept
H0: NY is not a Granger cause of CK	0.9521	accept	H0: CK is not a Granger cause of NY	0.6292	accept
H0: NY is not a Granger cause of NK	0.581	accept	H0: NK is not a Granger cause of NY	0.3238	accept
H0: NY is not a Granger cause of C	0.7368	accept	H0: C is not a Granger cause of NY	0.0182	reject
H0: NY is not a Granger cause of CE	0.0036	reject	H0: CE is not a Granger cause of NY	0.775	accept
H0: NY is not a Granger cause of NE	0.0491	reject	H0: NE is not a Granger cause of NY	0.7185	accept
H0: NY is not a Granger cause of NX	0.448	accept	H0: NX is not a Granger cause of NY	0.2442	accept
H0: NY is not a Granger cause of GX	0.6102	accept	H0: GX is not a Granger cause of NY	0.6152	accept
H0: NY is not a Granger cause of ZX	0.4889	accept	H0: ZX is not a Granger cause of NY	0.9518	accept
H0: NY is not a Granger cause of SX	0.7899	accept	H0: SX is not a Granger cause of NY	0.4056	accept
H0: NY is not a Granger cause of LS	0.487	accept	H0: LS is not a Granger cause of NY	0.6969	accept
H0: NY is not a Granger cause of NQ	0.1225	accept	H0: NQ is not a Granger cause of NY	0.017	reject
H0: NY is not a Granger cause of TQ	0.8464	accept	H0: TQ is not a Granger cause of NY	0.4331	accept
H0: NY is not a Granger cause of RDJ	0.5593	accept	H0: RDJ is not a Granger cause of NY	0.1041	accept
H0: NY is not a Granger cause of RDG	0.9671	accept	H0: RDG is not a Granger cause of NY	0.075	accept
H0: NY is not a Granger cause of K	0.1481	accept	H0: K is not a Granger cause of NY	0.5904	accept
H0: NY is not a Granger cause of ZS	0.6361	accept	H0: ZS is not a Granger cause of NY	0.4881	accept
H0: NY is not a Granger cause of R	0.2707	accept	H0: R is not a Granger cause of NY	0.8995	accept
H0: NY is not a Granger cause of CL	0.7401	accept	H0: CL is not a Granger cause of NY	0.2311	accept
H0: NY is not a Granger cause of S	0.3272	accept	H0: S is not a Granger cause of NY	0.6884	accept
H0: NY is not a Granger cause of H	0.6563	accept	H0: H is not a Granger cause of NY	0.8113	accept

**Table A5:** Results of Basic Statistical Analysis (h = *P*- value).

Variable	Skewness	Kurtosis	Jarque-Bera Test	Kolmogorov-Smirnov Test	Lilliefors Test
NY	−0.2345	3.1754	0.5000 (h = 0)	0.9979 (h = 0)	0.5000 (h = 0)
G	0.7501	2.8002	0.1388 (h = 0)	0.2844 (h = 0)	0.0179 (h = 1)
GZ	−1.0617	3.9518	0.0386 (h = 1)	0.7958 (h = 0)	0.3890 (h = 0)
Z	−0.6525	2.2081	0.1350 (h = 0)	0.4808 (h = 0)	0.0852 (h = 0)
N	−0.7102	4.1108	0.0820 (h = 0)	0.9077 (h = 0)	0.5000 (h = 0)
RG	0.4336	2.5821	0.5000 (h = 0)	0.4841 (h = 0)	0.0870 (h = 0)
GF	0.2139	4.2316	0.2404 (h=0)	0.0804 (h=0)	1.0000e-03 (h=1)
CK	0.4190	2.5406	0.5000 (h = 0)	0.5875 (h = 0)	0.1552 (h = 0)
NK	0.4754	2.7239	0.5000 (h = 0)	0.9122 (h = 0)	0.5000 (h = 0)
C	0.2885	2.6789	0.5000 (h = 0)	0.6120 (h = 0)	0.1751 (h = 0)
CE	−0.4257	4.1747	0.1608 (h = 0)	0.4816 (h = 0)	0.0857 (h = 0)
NE	0.7354	3.0702	0.1525 (h = 0)	0.6530 (h = 0)	0.2154 (h = 0)
NX	−2.8258	12.2786	1.0000e-03 (h = 1)	0.0038 (h = 1)	1.0000e-03 (h = 1)
GX	−0.3581	2.7356	0.5000 (h = 0)	0.9990 (h = 0)	0.5000 (h = 0)
ZX	−0.3776	2.6296	0.5000 (h = 0)	0.6553 (h = 0)	0.2178 (h = 0)
SX	4.3762e-04	2.1186	0.5000 (h = 0)	0.9744 (h = 0)	0.5000 (h = 0)
LS	−1.0269	3.2104	0.0551 (h = 0)	0.4188 (h = 0)	0.0567 (h = 0)
NQ	0.0351	2.2933	0.5000 (h = 0)	0.9426 (h = 0)	0.5000 (h = 0)
TQ	0.3674	2.9017	0.5000 (h = 0)	0.9493 (h = 0)	0.5000 (h = 0)
RDJ	0.8262	5.8171	0.0120 (h = 0)	0.6854 (h = 0)	0.2500 (h = 0)
RDG	1.5848	6.8869	0.0017 (h = 1)	0.0392 (h = 1)	1.0000e-03 (h = 1)
K	0.3928	2.3182	0.4636 (h = 0)	0.7640 (h = 0)	0.3433 (h = 0)
ZS	−0.1235	2.0941	0.5000 (h = 0)	0.9297 (h = 0)	0.5000 (h = 0)
R	−0.2686	2.7324	0.5000 (h = 0)	0.6726 (h = 0)	0.2362 (h = 0)
CL	1.3493	4.6532	0.0136 (h = 1)	0.6438 (h = 0)	0.2056 (h = 0)
S	0.2220	2.3003	0.5000 (h = 0)	0.8580 (h = 0)	0.5000 (h = 0)
ER	−0.4115	3.5320	0.5000 (h = 0)	0.3176 (h = 0)	0.0254 (h = 1)

**Table A6:** Correlation Measure Results

Variable	Correlation Measure	Gaussian Copula	t-Copula	Clatyon Copula	Frank Copula	Gumbel Copula	Raw Data
NY&G	Kendall's Rank τ	−0.0759	−0.0523	7.2543e-07	−0.0600	0.0178	−0.0842
	Spearman's Rank ρ	−0.1137	−0.1084	1.0922e-06	−0.0899	0.1674	−0.1038
	Euclidean Distance	3.9913	4.0156	3.9963	3.9936	4.0011	
NY&GZ	Kendall's Rank τ	0.0903	−0.0097	0.0222	0.0399	0.1810	−0.0105
	Spearman's Rank ρ	0.1351	−0.0103	0.0334	0.0599	0.2663	0.0241
	Euclidean Distance	1.2457	1.2594	1.2388	1.2407	1.2715	
NY&Z	Kendall's Rank τ	0.0876	0.1444	0.0797	0.0577	0.1006	0.0632
	Spearman's Rank ρ	0.1311	0.0138	0.1195	0.0864	0.1499	0.0541
	Euclidean Distance	1.1940	1.2053	1.1865	1.1868	1.2119	
NY&N	Kendall's Rank τ	−0.0144	−0.0239	5.6385e-04	−0.0312	1.3575e-06	0.0211
	Spearman's Rank ρ	−0.0217	−0.0106	8.4891e-04	−0.0467	2.0510e-06	0.0271
	Euclidean Distance	0.5058	0.5057	0.5060	0.5057	0.5060	
NY&RG	Kendall's Rank τ	−0.0666	−0.0959	7.2543e-07	−0.0856	0.0239	−0.0526
	Spearman's Rank ρ	−0.0997	−0.1037	1.0922e-06	−0.1281	0.0360	−0.1098
	Euclidean Distance	3.7642	3.7842	3.7682	3.7649	3.7741	
NY&GF	Kendall's Rank τ	0.0894	0.1187	0.0838	0.1080	0.0706	0.0526
	Spearman's Rank ρ	0.1338	0.0879	0.1254	0.1615	0.1056	0.0421
	Euclidean Distance	0.0142	0.0135	0.0148	0.0143	0.0147	
NY&CK	Kendall's Rank τ	−0.1871	−0.1492	7.2543e-07	−0.1871	1.3575e-06	−0.1579
	Spearman's Rank ρ	−0.2776	−0.1083	1.0922e-06	−0.2780	2.0510e-06	−0.2030
	Euclidean Distance	4.6734	4.6969	4.6869	4.6769	4.6869	
NY&NK	Kendall's Rank τ	0.1196	0.2095	0.1503	0.2410	0.2647	0.1895
	Spearman's Rank ρ	0.1786	0.1925	0.2233	0.3557	0.3839	0.2526
	Euclidean Distance	2.8575	2.9026	2.8508	2.8637	2.9046	
NY&C	Kendall's Rank τ	0.0352	0.0678	7.2543e-07	0.0753	0.1386	0.0842
	Spearman's Rank ρ	0.0528	0.1042	1.0922e-06	0.1127	0.2053	0.1353
	Euclidean Distance	0.0212	0.0195	0.0249	0.0184	0.0141	
NY&CE	Kendall's Rank τ	−0.1258	−0.0834	7.2543e-07	−0.1367	1.3575e-06	−0.1158
	Spearman's Rank ρ	−0.1877	−0.1025	1.0922e-06	−0.2039	2.0510e-06	−0.1835
	Euclidean Distance	0.0176	0.0137	0.0124	0.0191	0.0124	
NY&NE	Kendall's Rank τ	−0.0204	−0.0195	7.2543e-07	−0.0137	0.0202	−0.0421
	Spearman's Rank ρ	−0.0306	−0.1037	1.0922e-06	−0.0205	0.0304	−0.0150
	Euclidean Distance	0.0082	0.0080	0.0076	0.0080	0.0073	
NY&NX	Kendall's Rank τ	−0.0585	−0.0849	0.0274	−0.0855	1.3575e-06	−0.1053
	Spearman's Rank ρ	−0.0877	−0.1047	0.0411	−0.1279	2.0510e-06	−0.1248
	Euclidean Distance	0.0091	0.0099	0.0101	0.0101	0.0095	
NY&GX	Kendall's Rank τ	−0.1033	−0.1774	7.2543e-07	−0.1811	1.3575e-06	−0.1579
	Spearman's Rank ρ	−0.1545	−0.1032	1.0922e-06	−0.2692	2.0510e-06	−0.2256
	Euclidean Distance	0.0217	0.0241	0.0249	0.0241	0.0249	
NY&ZX	Kendall's Rank τ	−0.1092	−0.1601	7.2543e-07	−0.1569	1.3575e-06	−0.1158
	Spearman's Rank ρ	−0.1632	−0.1043	1.0922e-06	−0.2337	2.0510e-06	−0.2331
	Euclidean Distance	0.0300	0.0351	0.0260	0.0340	0.0260	
NY&SX	Kendall's Rank τ	−0.0354	−0.0483	7.2543e-07	−0.0279	−0.0561	−0.0247
	Spearman's Rank ρ	−0.0530	−0.0382	1.0922e-06	−0.0419	−0.0840	−0.0165
	Euclidean Distance	0.0149	0.0159	0.0127	0.0143	0.0105	
NY&LS	Kendall's Rank τ	−0.0693	−0.1276	7.2543e-07	−0.1349	1.3575e-06	−0.0632
	Spearman's Rank ρ	−0.1038	−0.0148	1.0922e-06	−0.2013	2.0510e-06	−0.1353
	Euclidean Distance	0.0131	0.0191	0.0100	0.0194	0.0100	
NY&NQ	Kendall's Rank τ	−0.0513	−0.2328	7.2543e-07	−0.1728	0.0521	−0.1053
	Spearman's Rank ρ	−0.0769	−0.1836	1.0922e-06	−0.2571	0.0781	−0.1368
	Euclidean Distance	0.0152	0.0276	0.0172	0.0183	0.0224	
NY&TQ	Kendall's Rank τ	0.0603	0.0998	0.1428	0.0738	1.3575e-06	0.1158
	Spearman's Rank ρ	0.0903	0.1024	0.2124	0.1105	2.0510e-06	0.1489
	Euclidean Distance	0.0322	0.0286	0.0202	0.0310	0.0398	
NY&RDJ	Kendall's Rank τ	−0.1669	−0.1400	7.2543e-07	−0.1588	1.3575e-06	−0.0526
	Spearman's Rank ρ	−0.2481	−0.1098	1.0922e-06	−0.2366	2.0510e-06	−0.1323
	Euclidean Distance	0.0510	0.0423	0.0283	0.0506	0.0283	
NY&RDG	Kendall's Rank τ	−0.0237	−0.0087	7.2543e-07	0.0470	1.3575e-06	0.0632
	Spearman's Rank ρ	−0.0355	−0.0103	1.0922e-06	0.0704	2.0510e-06	0.0782
	Euclidean Distance	0.0213	0.0216	0.0220	0.0247	0.0220	
NY&K	Kendall's Rank τ	−0.0136	0.0637	7.2543e-07	−0.0285	0.1569	−0.0316
	Spearman's Rank ρ	−0.0204	0.1038	1.0922e-06	−0.0427	0.2318	−0.0421
	Euclidean Distance	0.0235	0.0367	0.0244	0.0244	0.0512	
NY&ZS	Kendall's Rank τ	−0.1532	−0.2551	7.2543e-07	−0.1968	1.3575e-06	−0.1368
	Spearman's Rank ρ	−0.2282	−0.2049	1.0922e-06	−0.2920	2.0510e-06	−0.2286
	Euclidean Distance	0.0231	0.0355	0.0179	0.0286	0.0179	
NY&R	Kendall's Rank τ	−0.0048	−0.0546	7.2543e-07	−0.0739	0.1107	−0.0526
	Spearman's Rank ρ	−0.0072	−0.1093	1.0922e-06	−0.1108	0.1646	−0.1008
	Euclidean Distance	0.0065	0.0107	0.0064	0.0109	0.0082	
NY&CL	Kendall's Rank τ	−0.0541	−0.0812	7.2543e-07	−0.0626	1.3575e-06	−0.0526
	Spearman's Rank ρ	−0.0811	−0.1037	1.0922e-06	−0.0938	2.0510e-06	−0.0662
	Euclidean Distance	0.0145	0.0165	0.0122	0.0151	0.0122	
NY&S	Kendall's Rank τ	−0.2093	−0.3030	7.2543e-07	−0.2698	1.3575e-06	−0.2526
	Spearman's Rank ρ	−0.3097	−0.2084	1.0922e-06	−0.3966	2.0510e-06	−0.3774
	Euclidean Distance	0.0134	0.0190	0.0253	0.0158	0.0253	
NY&ER	Kendall's Rank τ	0.0566	−0.0050	0.1952	−0.0144	1.3575e-06	−0.0053
	Spearman's Rank ρ	0.0848	−0.1038	0.2882	−0.0216	2.0510e-06	−0.0143
	Euclidean Distance	0.0238	0.0290	0.0212	0.0275	0.0264	
